# Network Pharmacology-Based Study to Uncover Potential Pharmacological Mechanisms of Korean Thistle (*Cirsium japonicum* var. *maackii* (Maxim.) Matsum.) Flower against Cancer

**DOI:** 10.3390/molecules26195904

**Published:** 2021-09-29

**Authors:** Ki-Kwang Oh, Md. Adnan, Dong-Ha Cho

**Affiliations:** Department of Bio-Health Convergence, College of Biomedical Science, Kangwon National University, Chuncheon 24341, Korea; nivirna07@kangwon.ac.kr (K.-K.O.); mdadnan1991.pharma@gmail.com (M.A.)

**Keywords:** Akt1, cancer, *C. maackii* flower, network pharmacology, PI3K-Akt signaling pathway, Urs-12-en-24-oic acid, 3-oxo, methyl ester

## Abstract

*Cirsium japonicum* var. *maackii* (Maxim.) Matsum. or Korean thistle flower is a herbal plant used to treat tumors in Korean folk remedies, but its essential bioactives and pharmacological mechanisms against cancer have remained unexplored. This study identified the main compounds(s) and mechanism(s) of the *C. maackii* flower against cancer via network pharmacology. The bioactives from the *C. maackii* flower were revealed by gas chromatography-mass spectrum (GC-MS), and SwissADME evaluated their physicochemical properties. Next, target(s) associated with the obtained bioactives or cancer-related targets were retrieved by public databases, and the Venn diagram selected the overlapping targets. The networks between overlapping targets and bioactives were visualized, constructed, and analyzed by RPackage. Finally, we implemented a molecular docking test (MDT) to explore key target(s) and compound(s) on AutoDockVina and LigPlot+. GC-MS detected a total of 34 bioactives and all were accepted by Lipinski’s rules and therefore classified as drug-like compounds (DLCs). A total of 597 bioactive-related targets and 4245 cancer-related targets were identified from public databases. The final 51 overlapping targets were selected between the bioactive targets network and cancer-related targets. With Kyoto Encyclopedia of Genes and Genomes (KEGG) pathway enrichment, a total of 20 signaling pathways were manifested, and a hub signaling pathway (PI3K-Akt signaling pathway), a key target (Akt1), and a key compound (Urs-12-en-24-oic acid, 3-oxo, methyl ester) were selected among the 20 signaling pathways via MDT. Overall, Urs-12-en-24-oic acid, 3-oxo, methyl ester from the *C. maackii* flower has potent anti-cancer efficacy by inactivating Akt1 on the PI3K-Akt signaling pathway.

## 1. Introduction

The definition of cancer is that normal cells are damaged via an aberrant endogenous process such as an abnormality during DNA replication or instability of the DNA sequence, which transforms into malignancy [1]. The DNA damage responses represent chronic inflammation via the immune signaling pathway, which results in accelerating tumorigenesis [2]. The damaged normal cells undergo cellular senescence, triggering secretion in the inflammatory cytokines leading to cellular mechanical disruption [3,4]. Because inflammation is a leading factor in causing pathological symptoms such as unknown severe pain, fatigue, and comorbidity in cancer patients, anti-inflammation strategies are thus key therapeutics [5,6]. Current anti-inflammatory agents for cancer treatment NSAIDs, including Cox-2, due to fewer adverse effects and a lower mortality rate [7]. Most commonly, anti-cancer agents aim to inhibit DNA replication and induce cancer cell death; however, cancer chemotherapeutics also attack healthy cells resulting in serious side effects such as nausea, vomiting, hair loss, and fatigue [8,9,10]. On the other hand, traditional herbal plants with innovative bioactives and secondary metabolites play an essential role as effective anti-inflammatory, anti-oxidant, or anti-cancer agents [11]. For instance, plant extracts (*Urtica membranaceae*, *Artemesia monosperma*, and *Origanum dayi Post*) in combination with anti-cancer drugs showed enhanced potency against specific cancer cell lines (lung, breast, colon, and prostate cancer) without exposing normal lymphocytes and fibroblasts to cytotoxicity [12]. Herbal-derived bioactives possess fewer unwanted side effects than chemotherapy, and have led to new clinical drugs such as taxol from *Taxus brevifolia* L., vincristine from *Catharanthus roseus* G. Don, and Epipodophyllotoxin from *Podophyllum peltatum* L. [12,13].

*C. maackii* is a perennial herbal plant, belonging to the family of *Compositae*, and is widely distributed in the mountainous areas of Korea, Japan, and China [14]. Furthermore, *Cirsium* species have been reported to have diverse pharmacological activities such as anti-oxidant, anti-inflammation, anti-cancer, and hepatoprotection effects [15,16,17,18]. Specifically, a *C. maackii* extract at a concentration of 200 µg/mL showed 36.89% inhibition against a breast cancer cell line (MDA-MB-231) [19]. Another study demonstrated that HepG2 cells treated with a MeOH extract of *C. maackii* have potent antioxidant efficacy against severe oxidant conditions [20]. Anti-inflammatory agents assist in protection against cancer development, thereby preventing cytokine storms [21]. It implies that anti-inflammatory compounds are important agonists to protect normal cells adjacent to tumor cells because they can block the overflow of cytokines. Until now, *C. maackii* flower compounds were identified by HPLC and had only been reported for anti-Alzheimer efficacy by inhibiting BACE1 [22]. Generally, the identification of polar and mid-polar compounds from extracts is based on HPLC due to its good separation capability [23,24]. From a different perspective, we utilized GC-MS analysis to discover lipophilic bioactives, which mainly act as drug-like compounds and uptake efficiently into the cells. Lipophilicity is a significant physicochemical parameter that influences membrane permeability and affinity [25]. More importantly, GC-MS, along with the molecular docking test (MDT) and ADME (Absorption, Distribution, Metabolism, and Excretion) study, is an optimal analytical method to determine drug-like compounds [26]. At present, the bioactives and mechanisms of the *C. maackii* flower against cancer remain unknown. Hence, we aimed to uncover its potential bioactives with their fundamental mechanisms through network pharmacology.

Network pharmacology (NP) as a systemic method can analyze holistic bioactive–target–disease relationships [27]. It can decipher the unknown mechanism(s) with “multiple targets”, “multiple bioactives”, instead of “one target”, “one bioactive” [28]. This approach is a very effective method to recognize the mechanism of action for lead compounds discovered from herbal plants [29]. An existing drug may be re-modelled to bind on multiple targets with the concept of NP, thus it can be a guide for drug repurposing [30]. The NP application is a powerful tool to elucidate novel targets and bioactives from natural products, and is especially effective for anti-cancer research to investigate multi-target activity in biological pathways and interaction networks [31]. Here, we implemented NP to provide key bioactive(s), uppermost target(s), and potential mechanism(s) of the *C. maackii* flower against cancer. Mainly, the flower part of herbal plants are the most underexplored part compared to other parts such as leaves, roots, and stems. During the growing season of the *C. maackii* plant, the flowering part might be utilized as a source of essential bioactives or be taken as a functional food. In this study, we suggest that *C. maackii* flowers are valuable parts with potential anti-cancer compounds.

To prove their therapeutic value, we performed a GC-MS analysis, protein network investigation, and a molecular docking test (MDT). Its brief processes are discussed below.

**Figure 1:**
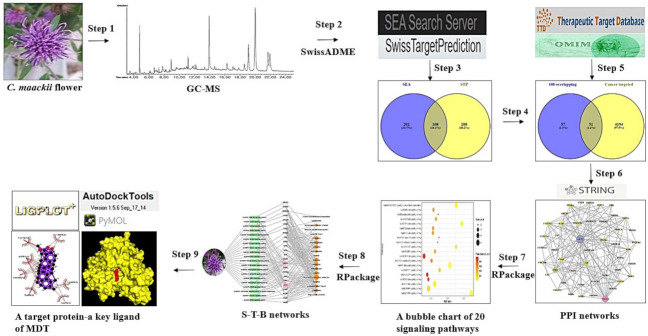
Workflow diagram of network pharmacology analysis of the *C. maackii* flower against cancer.

Firstly, bioactives from the *C. maackii* flower were identified by GC-MS analysis and screened to find drug-likeness compounds via an in silico tool. Then, targets associated with bioactives or cancer were identified through public databases, and final overlapping targets were utilized to analyze protein–protein interaction (PPI) and the highest degree of a target. Thirdly, a signaling pathway–target protein–bioactive (S–T–B) relationship against cancer was identified by networking analysis. Lastly, we found the most potent bioactive and a hub target to alleviate cancer severity by exploring the molecular mechanism of the *C. maackii* flower based on MDT. The workflow diagram is displayed in Figure 1.

## 2. Results

### 2.1. Bioactives from C. maackii Flower

**Figure 2:**
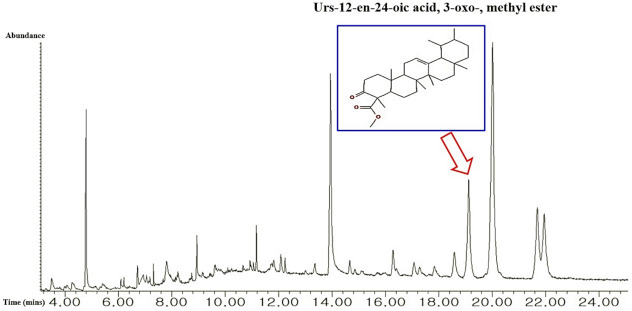
GC-MS peak of the *C. maackii* flower MeOH extract and an indication of the uppermost bioactive.

A total of 34 bioactives in the *C. maackii* flower were identified by GC-MS analysis (Figure 2), and the name of compounds, PubChem ID, retention time (mins), and peak area (%) are listed in Table 1. All 34 compounds were accepted by Lipinski’s rules (Molecular Weight ≤ 500 g/mol; Moriguchi octanol-water partition coefficient ≤ 4.15; Number of Nitrogen or Oxygen ≤ 10; Number of NH or OH ≤ 5), and all bioactives corresponded with the standard of “Abbott Bioavailability Score (>0.1)” through SwissADME. The TPSA (Topological Polar Surface Area) value of all bioactives was also accepted (Table 2).

### 2.2. Overlapping Targets between SEA and STP Associated with Bioactives

**Figure 3:**
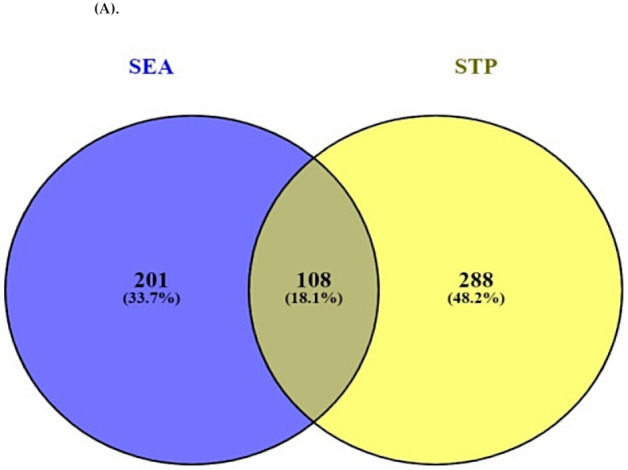

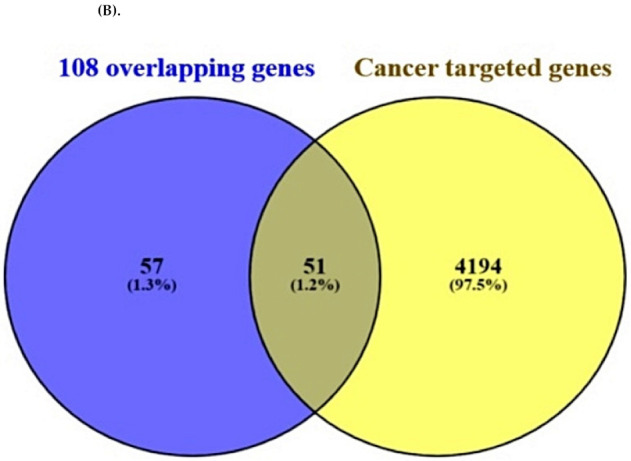
(**A**) Overlapping targets (108 targets) between SEA (309 targets) and STP (396 targets). (**B**) Overlapping targets (51 targets) between 108 overlapping targets from two databases (SEA and STP) and cancer associated with targets (4245 targets).

A total of 309 targets from SEA and 396 targets from STP connected to 34 bioactives were identified (Supplementary Table S1**)**. The Venn diagram showed that 108 targets overlapped between the two public databases (Supplementary Table S1) (Figure 3A).

### 2.3. Overlapping Targets between Cancer-Associated Targets and the Final 51 Overlapping Targets

A total of 4245 targets related to cancer were selected via retrieval from TTD and OMIM databases (Supplementary Table S2). The Venn diagram results revealed 51 overlapping targets that were selected between 4245 targets associated with cancer and 108 overlapping targets (Figure 3B) (Supplementary Table S3).

### 2.4. Acquisition of a Hub Target from PPI Networks

**Figure 4:**
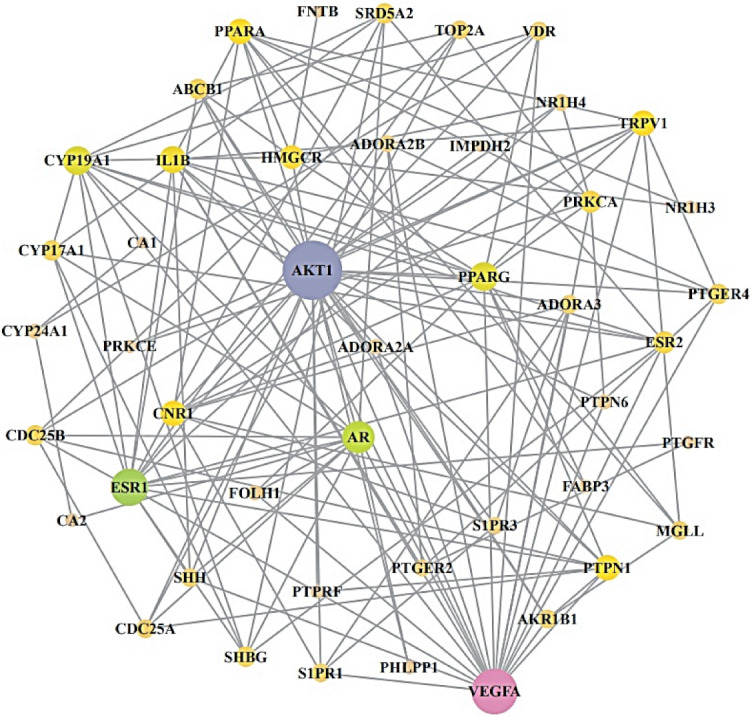
PPI networks (46 nodes, 145 edges). The size of circles represents the degree of values.

From STRING analysis, 46 out of 51 overlapping targets were directly related to cancer occurrence and development, indicating 46 nodes and 145 edges (Figure 4). The five targets removed (PAM, EPHX1, PPARD, KDM5C, and BCHE) had no connectivity to the overlapping 51 targets. In PPI networks, the Akt1 target was the highest degree (29) and was considered a hub target (Table 3).

### 2.5. Identification of a Hub Signaling from Bubble Chart

**Figure 5:**
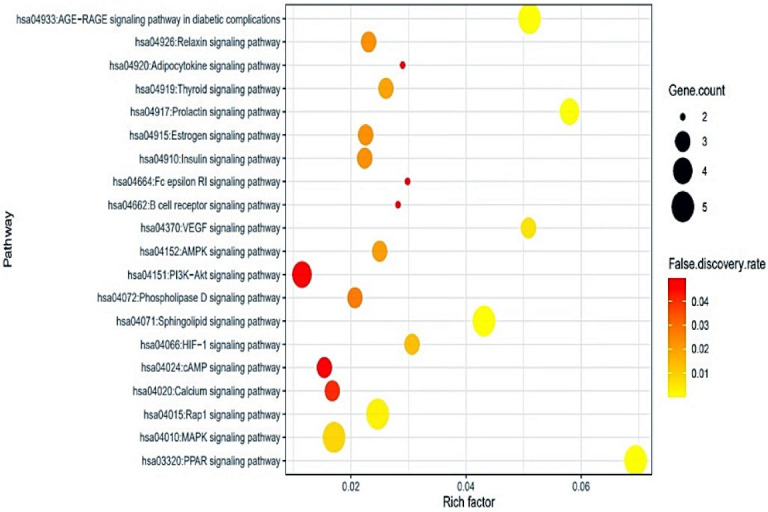
Bubble chart of 20 signaling pathways connected to cancer.

The output of KEGG (Kyoto Encyclopedia of Genes and Genomes) pathway enrichment analysis revealed that 20 signaling pathways were associated with 24 targets (False Discovery Rate < 0.05). The 20 signaling pathways were directly connected to cancer, suggesting that these 20 signaling pathways might be the noteworthy pathways of the *C. maackii* flower against cancer. The descriptions of 20 signaling pathways are displayed in Table 4. Additionally, a bubble chart suggested that the PI3K-Akt signaling pathway might be a hub signaling pathway of the *C. maackii* flower against cancer (Figure 5). Among the 20 signaling pathways, the Akt1 target was associated with 18 signaling pathways, representing the highest degree of value. Most importantly, Akt1 is directly related to the PI3K-Akt signaling pathway whereas both the PPAR signaling pathway and the Calcium signaling pathway are not correlated with Akt1.

### 2.6. S–T–B Network Analysis of C. maackii Flower against Cancer

**Figure 6:**
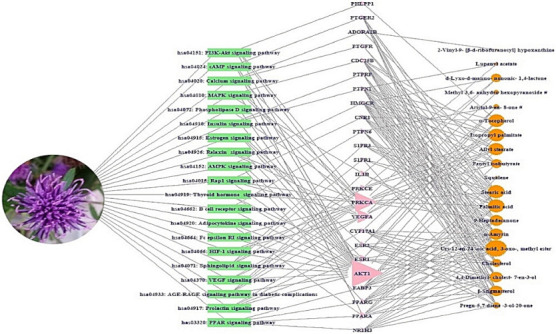
S–T–B networks (63 nodes, 204 edges). Green rectangle: Signaling pathway; pink triangle: Target protein; orange circle: Bioactive.

The S–T–B network of the *C. maackii* flower is exhibited in Figure 6. There were 20 pathways, 24 targets, and 19 bioactives (63 nodes and 204 edges). The nodes represented a total of three elements: Signaling pathways-Target protein-Bioactive (S-T-B). The edges represented relationships of a total number of three elements. The S–T–B relationship suggested that the network might interact with therapeutic efficacy against cancer. Regarding the 20 signaling pathways, the highest degree among targets was “Akt1” with 18 degrees, and the highest degree among bioactives was “Urs-12-en-24-oic acid, 3-oxo-, methyl ester” with 36 degrees (Table 5).

### 2.7. MDT of 3 Targets and 10 Bioactives Connected to PI3K-Akt Signaling Pathway

**Figure 7:**
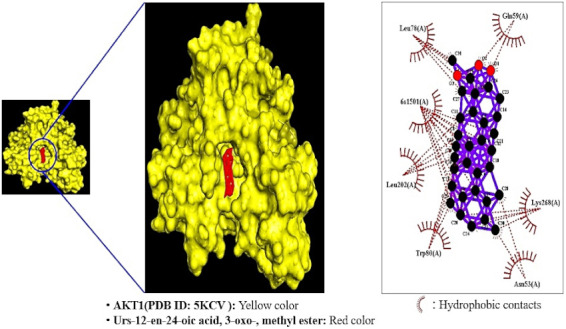
MDT of Urs-12-en-24-oic acid, methyl ester on Akt1 (PDB ID: 5KCV).

The Akt1 protein was related to two bioactives (Urs-12-en-24-oic acid, 3-oxo-, methyl ester and α-Tocopherol), the VEGFA target to three bioactives (Allyl stearate, Isopropyl palmitate, and Methyl 3,6-anhydro hexopyranoside #), and the PRKCA target with seven bioactives (Allyl stearate, Isopropyl palmitate, Pentyl isobutyrate, 9-Heptadecanone, Palmitic acid, Stearic acid, and d-Lyxo-d-manno-nononic- 1,4-lactone). MDT was performed to verify the affinity of target protein(s) and biactive(s), which displayed the docking figure of a hub target—the uppermost bioactive (Figure 7). The Akt1 protein (PDB ID: 5KCV) connected to two compounds on the PI3K-Akt signaling pathway was subjected to MDT. It was observed that Urs-12-en-24-oic acid, 3-oxo-, methyl ester (−12.8 kcal/mol) docked on the Akt1 protein (PDB ID: 5KCV) manifested the highest binding energy, followed by α-Tocopherol (−5.8 kcal/mol). The docking detail results of two bioactives are shown in Table 6. The MDT score of three bioactives on the VEGFA protein (PDB ID: 3V2A) was analyzed in the “Homo Sapiens” mode. It was revealed that Methyl 3,6-anhydro hexopyranoside # (−5.2 kcal/mol) docked on the VEGFA protein (PDB ID: 3V2A) exhibited the highest binding energy followed by Allyl stearate (−5.1 kcal/mol) and Isopropyl palmitate (−5.1 kcal/mol). The docking detail results of three bioactives are shown in Table 7. The MDT score of seven bioactives on the PRKCA protein (PDB ID: 3IW4) was analyzed in the “Homo Sapiens” mode. It was exposed that d-Lyxo-d-manno-nononic- 1,4-lactone (−6.9 kcal/mol) docked on the PRKCA protein (PDB ID: 3IW4) demonstrated the highest binding energy, followed by Isopropyl palmitate (−6.3 kcal/mol), Stearic acid (−6.2 kcal/mol), Allyl stearate (−6.2 kcal/mol), 9-Heptadecanone (−5.4 kcal/mol), Pentyl isobutyrate (−5.0 kcal/mol), and Palmitic acid (−4.8 kcal/mol). The docking detail results of seven bioactives are shown in Table 8. Collectively, both VEGFA (PDB ID: 3V2A) and PRKCA proteins (PDB ID: 3IW4) showed that the affinity of each bioactive did not give a valid binding score (>−7.0 kcal/mol) [45].

### 2.8. Comparative Analysis of MDT against Positive Controls on a Hub Target

A key bioactive (Urs-12-en-24-oic acid, 3-oxo-, methyl ester) associated with the PI3K-Akt signaling pathway revealed the greatest binding score (−12.8 kcal/mol) on Akt1 (PDB ID: 5KCV). The 13 positive controls on Akt1 (PDB ID: 5KCV) are as follows. The MDT score of BAY1125976 (PubChem ID: 70817911) was the highest score with −9.1 kcal/mol, followed by Miransertib (PubChem ID: 53262401; −9.0 kcal/mol)), Akti-1/2 (PubChem ID: 135398501; −8.9 kcal/mol), Oridonin (PubChem ID: 5321010; −8.3 kcal/mol), MK-2206 dihydrochloride (PubChem ID: 46930998; −7.8 kcal/mol), AT7867 (PubChem ID: 11175137; −7.6 kcal/mol), A-674563 (PubChem ID: 11314340; −7.6 kcal/mol), Gsk-690693 (PubChem ID: 16725726; −7.4 kcal/mol), Ipatasertib (PubChem ID: 24788740; −7.3 kcal/mol), Capivasertib (Pubchem ID: 25227436; −7.1 kcal/mol), AT13148 (PubChem ID: 24905401; −6.9 kcal/mol), Afuresertib (PubChem ID: 46843057; −6.9 kcal/mol), and Uprosertib (PubChem ID: 51042438; −6.8 kcal/mol), respectively. In particular, the MDT score of Urs-12-en-24-oic acid, 3-oxo-, methyl ester on Akt1 (PDB ID: 5KCV) had ahigher affinity (−12.8 kcal/mol) compared to the 13 positive controls. Detailed information is listed in Table 9.

### 2.9. Toxicological Properties of a Selected Key Bioactive

Additionally, the toxicological properties of Urs-12-en-24-oic acid, 3-oxo-, methyl ester were predicted by the admetSAR online tool. Our result suggested that the bioactive did not disclose Ames toxicity, carcinogenic properties, acute oral toxicity, or rat acute toxicity properties (Table 10).

## 3. Discussion

The S–T–B network suggested that the therapeutic efficacy of the *C. maackii* flower against cancer was directly associated with 20 signaling pathways, 24 targets, and 19 bioactives. Through the network, we identified the most significant protein (Akt1) associated with the occurrence and development of cancer and a bioactive (Urs-12-en-24-oic acid, 3-oxo-, methyl ester) from the *C. maackii* flower. From a bubble chart, we identified a hub signaling pathway (PI3K-Akt signaling pathway) connected to the Akt1 target, indicating the lowest rich factor among 20 signaling pathways. A report demonstrated that the activated PI3K-Akt signaling pathway accelerates tumor cell proliferation, invasion, and metastasis, inhibiting apoptosis [46]. Furthermore, it was found that the Akt1 target was overexpressed in 15 out of 24 human hepatocellular carcinomas (63.3%) confirmed using PCR analysis through Northern blot [47]. Another research study suggested that the PI3K-Akt signaling pathway’s antagonists are potential anti-cancer candidates to regulate acute and chronic inflammatory responses [48].

In the S–T–B network, Urs-12-en-24-oic acid, 3-oxo-, methyl ester had the highest degree of value and was considered the uppermost bioactive of the *C. maackii* flower against cancer. Urs-12-en-24-oic acid, 3-oxo-, methyl ester is categorized into boswellic acids used widely as anti-inflammatory agents, including as an anti-cancer treatment. Additionally, the boswellic acids have potent anti-cancer efficacy against diverse malignant cancers [49,50]. The KEGG pathway enrichment analysis of 24 targets suggested that a total of 20 signaling pathways were involved in cancer occurrence and development. The relationships of the 20 signaling pathways with cancer are concisely discussed as follows. In the Peroxisome Proliferator-Activated Receptor (PPAR) signaling pathway, the activation of the PPAR signaling pathway functions as an anti-inflammatory agent, which can overwhelm the metabolic energy balance of cancer cells by inhibiting the fatty acid synthesis and accelerating fatty acid oxidation [51,52]. In the Mitogen-Activated Protein Kinase (MAPK) signaling pathway, MAPK inhibitors are efficient blockers to reduce pro-inflammatory cytokines and enhance the anti-cancer effect, particularly on human pancreatic cancer cells [53,54]. In the Rap1 (Ras-associated protein-1) signaling pathway, Rap1 promotes cytokine production during the inflammatory condition, which leads to tumor progression in human colorectal cancer [55,56]. Regarding the alcium signaling pathway, calcium is a significant second messenger to regulate inflammation; hence, blockers of the calcium channel can induce cancer cell death [57,58]. In the Cyclic AMP (cAMP) signaling pathway, an increased cAMP level has an anti-inflammatory effect, where the increased level can regulate DNA damage, DNA repair, and apoptosis of cancer cells [59,60]. Concerning the Hypoxia-Inducible Factor-1 (HIF-1) signaling pathway, HIF-1 is a central regulator to stimulate the production of inflammation. HIF-1 overexpression is related to increased tumor growth [61,62]. In the sphingolipid signaling pathway, sphingolipid is implicated in the inflammatory response, which has been involved in cancer cell proliferation [63,64]. In the Phospholipase D (PLD) signaling pathway, PLD inhibition can induce two functions, namely anti-inflammation and anti-cancer. Mainly, the blocking of PLD during chemotherapy can sensitize one to chemotherapeutics [65,66]. Regarding the Phosphoinositide 3-kinase-Akt (PI3K-Akt) signaling pathway, inhibition of the PI3K-Akt signaling pathway reduces the severity of inflammation in mice. This strategy is a promising mechanism for the treatment of cancers such as lung cancer, colorectal cancer, renal cancer, prostate cancer, triple-negative breast cancer, mucinous adenocarcinoma of the ovary, and skin cancer [67,68]. In the AMP-activated protein kinase (AMPK) signaling pathway, AMPK activation suppresses inflammatory responses and dampens cancer growth with cell metabolism and the cell cycle [69,70]. Regarding the Vascular Endothelial Growth Factor (VEGF) signaling pathway, VEGF is a core mediator in the formation of new blood vessels for cancer cells; thereby, cancer cells can survive, grow, and metastasize [71]. In the B cell receptor (BCR) signaling pathway, B cells are involved in the inflammatory T cell receptor, which is implicated in antibody production [72]. B cells or BCR-associated kinases may function with anti-cancer activity via B cells activation [73]. In the Fc epsilon RI signaling pathway, the expression of Fc epsilon on mast cells stimulates immunoglobulin E, leading to type 1 hypersensitivity-induced local inflammatory responses at the tumor sites [74]. In the insulin signaling pathway, insulin-resistant patients undergo excessive production of reactive oxygen species (ROS) that can harm DNA attributed to carcinogenesis [75]. In the estrogen signaling pathway, estrogen exposure to chronic inflammatory disease activity is a key risk in breast cancer progression [76]. With regards to the prolactin signaling pathway, prolactin functions as a cytokine immune system, especially in breast cancer, which has the strongest correlation with an increased expression level of prolactin and prolactin receptors [77,78]. In the thyroid signaling pathway, the optimal regulation of the cellular thyroid hormone is essential for an adequate role of immune cells during inflammation [79]. In terms of the adipocytokine signaling pathway, adipocytokine is an inflammatory mediator during immune-associated diseases, which accelerates cancer progression and metastasizes from organ to organ [80]. In the Relaxin signaling pathway, Relaxin alleviates the inflammatory severity and diminishes the amount of leucocytes and the expression level of cytokines [81]. In terms of the Advanced Glycation End-product (AGE)-Advanced Glycation End-product Receptor (RAGE) signaling pathway in diabetic complications, RAGE can stimulate inflammatory responses by binding with AGEs [82]. The inhibitor (papaverine) of RAGE is a promising target for anti-cancer activity, which blocks nuclear factor kappa B (NF- κB) [83].

## 4. Materials and Methods

### 4.1. Plant Material Collection and Identification

The *C. maackii* flowers were collected from Mihogil of Bomunmyeon (Latitude: 36.666149, Longitude: 128.511759), Kyeongsang-bukdo, Republic of Korea, in October 2020, and the plant was identified by Dr. Dong Ha Cho, Plant biologist and Professor, Department of Bio-Health Convergence, College of Biomedical Science, Kangwon National University. A voucher number (CNB 015) has been deposited at the Kenaf Corporation in the Department of Bio-Health Convergence, and the material can only be used as research.

### 4.2. Plant Preparation, Extraction

The *C. maackii* flower was dried in a shady area at room temperature (20–22 °C) for 7 days, and dried leaves were powdered using an electric blender (Shinil, Cheonan, Korea). Approximately 50 g of *C. maackii* flower powder was soaked in 800 mL of 100% methanol (Daejung, Seohaean, Korea) for 5 days and repeated 3 times to collect extraction. The solvent extract was collected, filtered, and evaporated using a vacuum evaporator (RV8, IKA, Staufen, German). The evaporated sample was dried under a boiling water bath (HB10, IKA, Staufen, German) at 40 °C to obtain the extract.

### 4.3. GC-MS Analysis Condition

Agilent 7890A (Agilent, Santa Clara, CA, USA) was used to carry out the GC-MS analysis. GC was equipped with a DB-5 (30 m × 0.25 mm × 0.25 μm) capillary column (Agilent, Santa Clara, CA, USA). Initially, the instrument was maintained at a temperature of 100 °C for 2.1 min. The temperature rose to 300 °C at a rate of 25 °C/min and was maintained for 20 min. The injection port temperature and helium flow rate were sustained at 250 °C and 1.5 mL/min, respectively. The ionization voltage was 70 eV. The samples were injected in the split mode at 10:1. The MS scan range was set at 35–900 (*m/z*). The fragmentation patterns of mass spectra were compared with those stored in the W8N05ST Library MS database. The percentage of each compound was calculated from the relative peak area of each compound in the chromatogram. The concept of integration was used with the ChemStation integrator (Agilent, Santa Clara, CA, USA) algorithms (analyzed 19 May 2021) [84].

### 4.4. Bioactives Database Construction and Drug-Likeness Property

The bioactives from the *C. maackii* flower were identified by utilizing GC-MS analysis. Then, the GC-MS-detected bioactives were filtered in accordance with Lipinski’s rules through SwissADME (http://www.swissadme.ch/) (accessed on 3 June 2021) to confirm the “Drug-likeness” physicochemical properties. PubChem (https://pubchem.ncbi.nlm.nih.gov/) (accessed on 3 June 2021) was utilized to select the SMILES (Simplified Molecular Input Line Entry System) bioactives.

### 4.5. Target Targets Related to Selected Bioactives or Cancer

Targets connected to the bioactives were selected through both the Similarity Ensemble Approach (SEA) (http://sea.bkslab.org/) (accessed on 7 June 2021) [85] and Swiss Target Prediction (STP) (http://www.swisstargetprediction.ch/) (accessed on 9 June 2021) [86] with the “Homo Sapiens” setting, both of which are based on SMILES. The cancer-related targets in humans were obtained with keywords (cancer/tumor/neoplasia/carcinoma) from TTD (http://db.idrblab.net/ttd/) (accessed on 12 June 2021) and OMIM (https://www.omim.org/) (accessed on 13 June 2021). The overlapping targets between compounds of the *C. maackii* flower and cancer targets were illustrated by VENNY 2.1 (https://bioinfogp.cnb.csic.es/tools/venny/) (accessed on 14 June 2021).

### 4.6. Construction of PPI Networks and Bubble Chart

For the final overlapping targets, STRING (https://string-db.org/) (accessed on 16 June 2021) [87] was utilized to analyze the PPI network. Thereby, RPackage was used to identify the degree of value. Then, signaling pathways on STRING were visualized by RPackage, a hub signaling pathway (lowest rich factor) related to a hub target (highest degree of value from PPI).

### 4.7. Construction of a Size Map on S-T-B Network

Both the hub target (the highest degree of value among 20 signaling pathways) and the uppermost bioactive were identified via the S–T–B network. The S–T–B networks were utilized to construct a size map, based on the degree of values. In this size map, green rectangles (nodes) represented signaling pathways, pink triangles (nodes) represented targets, and orange circles (nodes) represented bioactives. The circle size represented the degree value, the size of pink triangles represented the number of connectivity with signaling pathways, and the size of orange circles represented the number of connections with targets. The merged networks were constructed using RPackage.

### 4.8. Preparation for MDT of Targets

Four targets of a hub signaling pathway, i.e., Akt1 (PDB ID: 5KCV), VEGFA (PDB ID: 3V2A), PRKCA (PDB ID: 3IW4), and PHLPP1 (PDB ID: N/A), were selected on STRING via RCSB PDB (https://www.rcsb.org/) (accessed on 19 June 2021). Specifically, the PHLPP1 protein structure was not determined; thereby, the PDB ID was not uploaded in the .pdb format. Thus, the final three proteins selected in the .pdb format were converted into the .pdbqt format via Autodock (http://autodock.scripps.edu/) (accessed on 22 June 2021) [88].

### 4.9. Preparation for MDT of Positive Standard Ligands

A total of 13 positive control compounds on Akt1 antagonists, i.e., MK-2206 dihydrochloride (PubChem ID: 46930998), Gsk-690693 (PubChem ID: 16725726), Ipatasertib (PubChem ID: 24788740), Capivasertib (PubChem ID: 25227436), AT7867 (PubChem ID: 11175137), A-674563 (PubChem ID: 11314340), Miransertib (PubChem ID: 53262401), BAY1125976 (PubChem ID: 70817911), Akti-1/2 (PubChem ID: 135398501), Uprosertib (PubChem ID: 51042438), Afuresertib (PubChem ID: 46843057), AT13148 (PubChem ID: 24905401), and Oridonin (PubChem ID: 5321010), were selected to verify the docking score.

### 4.10. Preparation for MDT of Ligand Molecules

The ligand molecules were converted from sdf in PubChem into the .pdb format using Pymol, and the ligand molecules were converted into the .pdbqt format through Autodock.

### 4.11. Ligand-Protein Docking

The ligand molecules were docked with targets utilizing autodock4 by setting up an energy range of 4 and exhaustiveness at 8 as the default to obtain 10 different positions of ligand molecules [89]. The center (the position of the middle coordinate point) in the target was X: −12.677, Y: 2.931, Z: −13.145 on AKT1 (PDB ID: 5KCV). The grid box size was set to 40 Å × 40 Å × 40 Å. The 2D binding interactions were used with LigPlot+ v.2.2 (https://www.ebi.ac.uk/thornton-srv/software/LigPlus/) (accessed on 23 June 2021) [90]. After docking, ligands of the lowest binding energy (highest affinity) were selected to visualize the ligand–protein interaction in Pymol.

### 4.12. Toxicological Properties Prediction by admetSAR

Toxicological properties of the key bioactive were established using the admetSAR web-service tool (http://lmmd.ecust.edu.cn/admetsar1/predict/) (accessed on 24 June 2021) [91] because toxicity is an essential factor in developing new drugs. Hence, Ames toxicity, carcinogenic properties, acute oral toxicity, and rat acute toxicity were predicted by admetSAR.

## 5. Conclusions

The bioactives and mechanisms of *C. maackii* flowers against cancer were firstly uncovered through network pharmacology. The findings suggested that 20 signaling pathways, 24 targets, and 19 bioactives are connected to cancer. Of these, the PI3K-Akt signaling pathway, Akt1, and Urs-12-en-24-oic acid, 3-oxo-, methyl ester were the hub signaling pathway, hub target, and key bioactive of *C. maackii* flowers against cancer, respectively. Furthermore, Urs-12-en-24-oic acid, 3-oxo-, methyl ester has the most potent efficacy on the Akt1 target protein than 13 other standard ligands. This study suggests that the mechanism of the *C. maackii* flower against cancer might strengthen anti-inflammatory responses by inactivating the PI3K-Akt signaling pathway, bound to Urs-12-en-24-oic acid, 3-oxo-, methyl ester on Akt1. From this viewpoint, we propose that *C. maackii* flowers can be utilized as functional or medicinal resources against cancer.

## Figures and Tables

**Table 1:** A list of the 34 bioactives identified from *C. maackii* through GC-MS and profiling of bioactivities.

No.	Compounds	Pubchem ID	RT (mins)	Area (%)	Pharmacological Activities (References)
1	Glyceraldehyde	751	3.510	0.91	No reported
2	6-Methyluracil	12283	4.279	0.88	No reported
3	3-Hydroxy-2,3-dihydromaltol	119838	4.789	5.19	No reported
4	Pyranopyridine	534033	5.414	0.69	No reported
5	Formicin	69365	6.096, 6.212	0.58	No reported
6	4-Nitro-2-picoline *N*-oxide	95291	6.721	0.6	No reported
7	2-Vinyl-9-[.beta.-d-ribofuranosyl]hypoxanthine	135493011	6.933	1.35	Antibacterial and antifungal activity [32]
8	2-Chloromethyl-3,3-dichloropropene	43492	7.058	0.51	No reported
9	d-Lyxo-d-manno-nononic-1,4-lactone	535556	7.183	0.43	No reported
10	Pentyl isobutyrate	75554	7.327	0.26	Antimicrobial activity [33]
11	2-Methyl-3-nitrosooxazolidine	38357	7.673	0.23	No reported
12	*N*-Nitrosomethylethanolamine	33646	7.808	2.71	No reported
13	1,4,7,10-Tetraoxacyclododecan-2-one	533646	8.145	0.15	No reported
14	4-methylpyrimidin-2-ol	407091	8.231	0.58	No reported
15	Diethyl malonate	7761	8.750	0.46	No reported
16	Palmitic acid	985	8.943, 9.443, 9.760, 10.106	4.03	Anti-inflammation [34]
17	Methyl 3,6-anhydrohexopyranoside #	91691384	9.154	0.65	No reported
18	3-Hydroxypropionic acid	68152	9.625	1.17	Antibacterial activity [35]
19	2,2,4-Trichloro-1,3-cyclopentenedione	150757	9.846	0.65	No reported
20	Isopropyl palmitate	8907	10.673	0.54	Antibacterial activity [36]
21	Stearic acid	5281	10.933	0.72	Antibacterial activity [37]
22	Pregn-5,7-diene-3-ol-20-one	21117403	11.068	0.25	No reported
23	9-Heptadecanone	10887	11.173	0.95	Antibacterial activity [38]
24	Allyl stearate	80500	12.087	1.05	No reported
25	Squalene	638072	12.250	0.54	Anti-oxidant [39]
26	Prexanthoperol	628742	13.952	13.65	No reported
27	α-Tocopherol	14985	14.664	0.94	Anticancer [40]
28	β-Stigmasterol	6432745	16.289	1.68	Anti-inflammation [39]
29	4,4-Dimethylcholest-7-en-3-ol	5460076	17.077	1.06	No reported
30	Aristol-9-en-8-one #	6432651	17.837	1.11	No reported [39]
31	α-Amyrin	73170	18.587	2.53	Anti-obesity [41]
32	Urs-12-en-24-oic acid, 3-oxo-, methyl ester	612822	19.125	9.04	Potential Anti-inflammation [42]
33	Lupenyl acetate	323074	20.019	24.42	Antibacterial activity [43]
34	Cholesterol	5997	21.693, 21.952	15.19	Protective cell against pathogens [44]

PCIDB: PhytoChemical Interactions DB.

**Table 2:** Physicochemical properties of bioactives for good oral bioavailability and cell membrane permeability.

	Compounds	Lipinski Rules				Lipinski’s Violations	Bioavailability Score	TPSA (Å^2^)
		MW	HBA	HBD	MLog P			
No.		<500	<10	≤5	≤4.15	≤1	>0.1	<140
1	Glyceraldehyde	90.08	3	2	−1.66	0	0.55	57.53
2	6-Methyluracil	126.11	2	2	−0.39	0	0.55	65.72
3	3-Hydroxy-2,3-dihydromaltol	144.13	4	2	−1.77	0	0.85	66.76
4	Pyranopyridine	133.15	2	0	0.73	0	0.55	22.12
5	Formicin	89.09	2	2	−0.85	0	0.55	49.33
6	4-Nitro-2-picoline *N*-oxide	154.12	3	0	−0.15	0	0.55	71.28
7	2-Vinyl-9-[.beta.-d-ribofuranosyl]hypoxanthine	294.26	7	4	−1.77	0	0.55	133.49
8	2-Chloromethyl-3,3-dichloropropene	159.44	0	0	2.81	0	0.55	0.00
9	d-Lyxo-d-manno-nononic-1,4-lactone	268.22	9	7	−3.85	1	0.55	167.91
10	Pentyl isobutyrate	158.24	2	0	2.28	0	0.55	26.30
11	2-Methyl-3-nitrosooxazolidine	116.12	3	0	−0.42	0	0.55	41.90
12	*N*-Nitrosomethylethanolamine	104.11	3	1	−0.89	0	0.55	52.90
13	1,4,7,10-Tetraoxacyclododecan-2-one	190.19	5	0	−0.99	0	0.55	53.99
14	4-methylpyrimidin-2-ol	110.11	2	1	−0.27	0	0.55	45.75
15	Diethyl malonate	160.17	4	0	0.6	0	0.55	52.60
16	Palmitic acid	256.42	2	1	4.19	1	0.85	37.30
17	Methyl 3,6-anhydrohexopyranoside #	176.17	5	2	−1.59	0	0.55	68.15
18	3-Hydroxypropionic acid	90.08	3	2	−0.85	0	0.85	57.53
19	2,2,4-Trichloro-1,3-cyclopentenedione	199.42	2	0	0.55	0	0.55	34.14
20	Isopropyl palmitate	298.5	2	0	4.91	1	0.55	26.30
21	Stearic acid	284.48	2	1	4.67	1	0.85	37.30
22	Pregn-5,7-diene-3-ol-20-one	314.46	2	1	3.95	0	0.55	37.30
23	9-Heptadecanone	254.45	1	0	4.55	1	0.55	17.07
24	Allyl stearate	324.54	2	0	5.25	1	0.55	26.30
25	Squalene	410.72	0	0	7.93	1	0.55	0.00
26	Prexanthoperol	314.42	3	1	2.94	0	0.55	54.37
27	Vitamin E	430.71	2	1	6.14	1	0.55	29.46
28	β-Stigmasterol	412.69	1	1	6.62	1	0.55	20.23
29	4,4-Dimethylcholest-7-en-3-ol	414.71	1	1	6.73	1	0.55	20.23
30	Aristol-9-en-8-one #	218.33	1	0	3.56	0	0.55	17.07
31	α-Amyrin	426.72	1	1	6.92	1	0.55	20.23
32	Urs-12-en-24-oic acid, 3-oxo-, methyl ester	468.71	3	0	5.92	1	0.55	43.37
33	Lupenyl acetate	144.88	2	0	7.08	1	0.55	26.30
34	Cholesterol	386.65	1	1	6.34	1	0.55	20.23

MW, Molecular Weight (g/mol); HBA, Hydrogen Bond Acceptor; HBD, Hydrogen Bond Donor; MLog P, Lipophilicity; Bioavailability Score, the ability of a drug or other substance to be absorbed and used by the body.

**Table 3:** The degree value of PPI networks.

No.	Target	Degree	No.	Target	Degree
1	AKT1	29	24	MGLL	5
2	VEGFA	21	25	S1PR1	5
3	ESR1	16	26	SHH	5
4	AR	13	27	VDR	5
5	CYP19A1	11	28	TOP2A	5
6	PPARG	11	29	ADORA2B	4
7	IL1B	10	30	PTGER2	4
8	CNR1	9	31	S1PR3	4
9	TRPV1	9	32	ADORA2A	3
10	PPARA	9	33	CYP24A1	3
11	PTPN1	9	34	FOLH1	3
12	HMGCR	8	35	PTPN6	3
13	ESR2	7	36	CA2	2
14	PRKCA	7	37	FABP3	2
15	ABCB1	6	38	NR1H3	2
16	CDC25B	6	39	PHLPP1	2
17	PTGER4	6	40	PTPRF	2
18	SHBG	6	41	PRKCE	2
19	CYP17A1	6	42	PTGFR	2
20	SRD5A2	6	43	CA1	1
21	ADORA3	5	44	FNTB	1
22	AKR1B1	5	45	NR1H4	1
23	CDC25A	5	46	IMPDH2	1

**Table 4:** The degree value of S–T–B networks.

No.	Target	Degree	Bioactive	Degree	No.	Target	Degree	Bioactive	Degree
1	AKT1	18	Urs-12-en-24-oic acid,3-oxo-, methyl ester	36	13	S1PR1	1	Cholesterol	14
2	PRKCA	12	Palmitic acid	27	14	S1PR3	1	Pregn-5,7-diene-3-ol-20-one	10
3	VEGFA	7	Stearic acid	26	15	PTPN6	1	Methyl 3,6-anhydro hexopyranoside #	7
4	PPARA	3	α-Tocopherol	26	16	CNR1	1	Aristol-9-en-8-one #	7
5	ESR1	3	Isopropyl palmitate	26	17	HMGCR	1	Squalene	3
6	PPARG	2	Allyl stearate	25	18	PTPN1	1	Lupenyl acetate	3
7	ESR2	2	α-Amyrin	21	19	PTPRF	1	2-Vinyl-9- [β-d-ribofuranosyl] hypoxanthine	1
8	PRKCE	2	9-Heptadecanone	21	20	CDC25B	1		
9	IL1B	2	β-Stigmasterol	20	21	PTGFR	1		
10	NR1H3	1	4,4-Dimethyl-cholest-7-en-3-ol	20	22	ADORA2B	1		
11	FABP3	1	Pentyl isobutyrate	17	23	PTGER2	1		
12	CYP17A1	1	d-Lyxo-d-manno-nononic-1,4-lactone	15	24	PHLPP1	1		

**Table 5:** Targets in 20 signaling pathways’ enrichment related to cancer.

KEGG ID & Description	Targets	False Discovery Rate
hsa03320: PPAR signaling pathway	PPARA, PPARD, PPARG, NR1H3, FABP3	0.0000695
hsa04917: Prolactin signaling pathway	AKT1, ESR1, ESR2, CYP17A1	0.00063
hsa04933: AGE-RAGE signaling pathway in diabetic complications	AKT1, VEGFA, PRKCA, PRKCE, IL1B	0.00018
hsa04370: VEGF signaling pathway	AKT1, VEGFA, PRKCA	0.0068
hsa04071: Sphingolipid signaling pathway	AKT1, VEGFA, PRKCE, S1PR1, S1PR3	0.00027
hsa04066: HIF-1 signaling pathway	AKT1, VEGFA, PRKCA	0.016
hsa04664: Fc epsilon RI signaling pathway	AKT1, PRKCA	0.0492
hsa04920: Adipocytokine signaling pathway	AKT1, PPARA	0.0492
hsa04662: B cell receptor signaling pathway	AKT1, PTPN6	0.0492
hsa04919: Thyroid signaling pathway	AKT1, ESR1, PRKCA	0.0236
hsa04015: Rap1 signaling pathway	AKT1, VEGFA, PRKCA, VEGFA, ADORA2B	0.003
hsa04152: AMPK signaling pathway	AKT1, PPARG, HMGCR	0.025
hsa04926: Relaxin signaling pathway	AKT1, VEGFA, PRKCA	0.0278
hsa04915: Estrogen signaling pathway	AKT1, ESR1, ESR2	0.0279
hsa04910: Insulin signaling pathway	AKT1, PTPN1, PTPRF	0.0279
hsa04072: Phospholipase D signaling pathway	AKT1, PRKCA	0.0324
hsa04010: MAPK signaling pathway	AKT1, VEGFA, PRKCA, IL1B	0.0103
hsa04020: Calcium signaling pathway	PRKCA, ADORA2B	0.0457
hsa04024: cAMP signaling pathway	AKT1, PPARA, PTGER2	0.0492
hsa04151: PI3K-Akt signaling pathway	AKT1, VEGFA, PRKCA, PHLPP1	0.0487

**Table 6:** Binding energy of potential bioactives on Akt1 (PDB ID: 5KCV).

				Hydrogen Bond Interactions			Hydrophobic Interactions
Protein	Ligand	PubChem ID	Binding Energy (kcal/mol)	Amino Acid Residue	R Group(s) Involved in Hydrogen Bonding	Distance (Å)	Amino Acid Residue
5KCV	Urs-12-en-24-oic acid, 3-oxo-, methyl ester	612822	−12.8	N/A	N/A	N/A	Gln59, Lys268, Asn53
							Trp80, Leu202, Leu78
	α-Tocopherol	14985	−5.8	Leu78	R-OH	2.71	Ala58, Val270, Lys268
							Val201, Leu202, Gln203
							Trp80, Asn53

**Table 7:** Binding energy of potential bioactives on VEGFA (PDB ID: 3V2A).

				Hydrogen Bond Interactions			Hydrophobic Interactions
Protein	Ligand	PubChem ID	Binding Energy (kcal/mol)	Amino Acid Residue	R Group(s) Involved in Hydrogen Bonding	Distance (Å)	Amino Acid Residue
3V2A	Allyl stearate	80500	−5.1	Phe47	RCOOR′	3.18	Ile46, Lys48, Lys286
							Asp276, Arg275, Pro40
							Phe36
	Isopropyl palmitate	8907	−5.1	N/A	N/A	N/A	Asp276, Asp34, Ile46
							Phe47, Lys48, Lys286
							Phe36, Pro40
	Methyl 3,6-anhydro hexopyranoside #	91691384	−5.2	Ser310, Pro85	R-O-R′, R-OH	2.98, 3.23, 2.82	Lys84, Gln87, Gly312
							Asp257, Gly255, Ile256
							Glu44

**Table 8:** Binding energy of potential bioactives on PRKCA (PDB ID: 3IW4).

					Hydrogen Bond Interactions		Hydrophobic Interactions
Protein	Ligand	PubChem ID	Binding Energy (kcal/mol)	Amino Acid Residue	R Group(s) Involved in Hydrogen Bonding	Distance (Å)	Amino Acid Residue
3IW4	Allyl stearate	80500	−6.2	Lys 396	RCOOR′	3.21	Asp395, Leu393, Pro397
							Arg608, Pro398, Val664
							Pro666, Ser473, Glu474
							His665, Lys478, Ile667
							Gln402, Asn660
	Isopropyl palmitate	8907	−6.3	Asn660	RCOOR′	2.92	Asp395, Leu393, Pro397
							Lys396, Gln402,Pro398
							Arg608, Ile667, Pro666
							Val664, His665, Lys478
				N/A	N/A	N/A	Pro502, Gln650, Ile645
	Pentyl isobutyrate	75554	−5.0				Gly540, Asp542, Asp503
							Asp539, Gln642, Leu546
							Glu543
	9-Heptadecanone	10887	−5.4	Lys478	RCOR′	2.80	Asn607, Gln548, Pro398
							Arg608, Ile667, Tyr419
							Asn421, Ser473, Pro666
							Glu418, Asp472, His665
							Val664, Gln402, Glu552
	Palmitic acid	985	−4.8	Asn660, Lys396	RCOR′, R-OH	2.93, 2.95	Val664, Pro398, Glu552
							Gln662, Asp395, Leu393
							Leu394, Gln402, Pro397
	Stearic acid	5281	−6.2	Lys396, Leu393	R-OH	3.04, 2.95	Leu394, Gln402, Pro398
							Lys478, His665, Ile667
							Pro666, Val664, Arg608
							Pro397, Asn660
	d-Lyxo-d-manno-nononic- 1,4-lactone	535556	−6.9	Leu393, Lys396, Pro397	RCOOR′, R-OH	3.30, 2.73, 3.31	Leu394, Asp395, Gln662
				Gln402		3.23, 3.19, 2.66	Asn660, Pro398
						3.08, 3.03, 3.15	
						3.26, 2.88	

**Table 9:** Binding energy of the positive controls on Akt1 (PDB ID: 5KCV).

					Hydrogen Bond Interactions		Hydrophobic Interactions
Protein	Ligand	PubChem ID	Binding Energy (kcal/mol)	Amino Acid Residue	R Group(s) Involved in Hydrogen Boding	Distance (Å)	Amino Acid Residue
5KCV	MK-2206 dihydrochloride	46930998	−7.8	N/A	N/A	N/A	Leu78, Leu202, Gln203
							Lys268, Trp80, Ala58
							Gln59
	Gsk-690693	16725726	−7.4	Tyr18, Asp274	RNR′,RNHH	3.18, 3.06	Glu85, Phe161, Val83
							Cys296, Gly294, Phe293
							Lys276, Leu316, Pro313
							Arg273, Leu295, Ile84
	Ipatasertib	24788740	−7.3	N/A	N/A	N/A	Lys268, Asn53, Gln79
							Ala58, Phe225, Leu223
							Leu202, Leu78, Trp80
	Capivasertib	25227436	−7.1	Val83	ROH	2.95	Ile84, Arg273, Asn279
							Asp274, Thr291, Glu278
							Phe293, Gly294, Cys296
							Glu85, Glu17
	AT7867	11175137	−7.6	Ser56	RNH	3.04	Lys268, Trp80, Asn53
							Leu78, Cys60, Gln79
							Ala58, Cys77, Val201
							Leu202, Gln203
	A-674563	11314340	−7.6	Gln79, Gln203	RNHH, RNH	3.16, 3.20	Ser56, Trp80, Leu202
							Lys268, Asn53, Ala58
							Leu78
	Miransertib	53262401	−9.0	Gln203, Leu78	RNHH	3.19, 3.22	Lys268, Trp80, Leu202
							Phe225, Ser216, Val201
							Gln59, Gln79, Ala58
	BAY1125976	70817911	−9.1	Asp274	RNHH	3.29	Gly294, Thr291, Glu278
							Asn279, Tyr229, Leu156
							Glu234, Phe293, Lys158
							Cys296
	Akti-1/2	135398501	−8.9	Cys296, Arg15	RCOR′, RNR′	3.03, 3.15, 3.19	Glu278, Gly294, Leu295
							Val83, Gln85, Glu17
							Tyr18, Ile84, Phe293
							Asn279
	Uprosertib	51042438	−6.8	Cys296, Leu295, Glu278	RCOR′, RCOR′, RNHH	2.93, 2.86, 2.93	Tyr18, Arg273, Asp274
							Lys276, Thr291, Phe293
							Asn279, Gly294, Ile84
							Thr82
	Afuresertib	46843057	−6.9	Gly394, Gly395	RNHH	3.10, 2.95	Ala50, Arg328, Lys389
							Pro388, Ala329, Asp325
							Gly327, Tyr326, Phe55
							Ile36, Leu52, Pro51
	AT13148	24905401	−6.9	Glu341, Tyr315, His354	ROR′, ROR′, RNR′	3.01, 2.70, 3.14	Phe236, Glu278, Leu347
							Pro313, Tyr350, Glu314
	Oridonin	5321010	−8.3	Asp325, Ala50, Arg328	RCOR′, ROH, ROH, ROH	2.67, 2.71, 2.80, 3.24	Phe55, Ile36, Leu52
							Gly394, Ala329, Gly327
							Tyr326

**Table 10:** Toxicological properties of the uppermost bioactive on AKT1 (PDB ID: 5KCV) in MDT.

Parameters	Compound Name
	Urs-12-en-24-oic acid, 3-oxo-, Methyl Ester
Ames toxicity	NAT
Carcinogens	NC
Acute oral toxicity	Ⅲ
Rat acute toxicity	2.1675

AT: Ames toxic; NAT: Non-Ames toxic; NC: Non-carcinogenic; Category-II means (50 mg/kg > LD50 < 500 mg/kg); Category-III means (500 mg/kg > LD50 < 5000 mg/kg).

## Data Availability

All data generated or analyzed during this study are included in this published article (and its Supplementary Information Files).

## Supplementary Materials

The following are available online, Table S1: The 309, 396, and 108 targets from SEA, STP, and overlapping targets between SEA and STP, respectively. Table S2: The 4245 cancer-related targets from TTD and OMIM databases, Table S3: The final 51 targets against cancer.

## Sample Availability

Samples of described compounds are available from the authors.

## Abbreviations

ADME	Absorption: Distribution, Metabolism, Excretion;
AGE	Advanced Glycation End-product;
AMPK	AMP-activated protein kinase;
cAMP	Cyclic AMP;
*C. maackii*	*Cirsium japonicum* var. *maackii* (Maxim.) Matsum.: *C. maackii*;
DLCs	Drug-Like Compounds;
BCR	B cell receptor;
GC-MS	Gas Chromatography Mass Spectrum;
HIF-1	Hypoxia Inducible Factor-1;
KEGG	Kyoto Encyclopedia of Genes and Genomes;
MAPK	Mitogen Activated Protein Kinase;
MDT	Molecular Docking Test;
NF- κB	Nuclear Factor Kappa B;
OMIM	Online Mendelian Inheritance in Man;
PI3K-Akt	Phosphoinositide 3-kinase-Akt;
PLD	Phospholipase D;
PPAR	Peroxisome Proliferator Activated Receptor;
PPI	Protein-protein interaction;
RAGE	Advanced Glycation End-product Receptor;
Rap1	Ras-associated protein-1;
ROS	Reactive Oxygen Species;
SEA	Similarity Ensemble Approach;
SMILES	Simplified Molecular Input Line Entry System;
S-T-B	Signaling pathway-Target protein-Bioactive;
STP	SwissTargetPrediction;
t-BHP	Tert-butyl hydroperoxide;
TTD	Therapeutic Target Database;
VEGF	Vascular Endothelial Growth Factor
