# Supplementary material for: Network Pharmacology-Based Study to Uncover Potential Pharmacological Mechanisms of Korean Thistle (Cirsium japonicum var. maackii (Maxim.) Matsum.) Flower against Cancer
Source: Molecules. 2021 Sep 29;26(19):5904. doi: 10.3390/molecules26195904 (PMC8513069; doi:10.3390/molecules26195904)
Supplement: Supplementary file 1 [file molecules-26-05904-s001.zip › Supplementary Table S1.pdf]

The number of 309 targets from SEA;	The number of 396 targets from STP;	The number of 108 overlapping targets between SEA and STP
AKR1B1	AKR1B1	AKR1B1
TPO	GBA	MTNR1B
HMBS	CYP2A6	MTNR1A
UNG	SIRT3	PTPRC
TYMP	SIRT2	CES2
GPR84	IDO1	ADORA3
ENGASE	TYR	ADORA1
MTNR1B	DAO	ADORA2A
MTNR1A	FUCA1	ADK
VCAM1	PRKCA	ADORA2B
DDX3X	PRKCD	IMPDH2
PHLPP2	TRPV4	ADH1A
NFE2L2	ABCB1	POLA1
SMARCA2	HMGCR	POLB
PTPRC	PRKCG	PAM
MBD2	PRKCE	FAAH
CASP4	PRKCH	EPHX1
CES2	PRKCQ	CES1
PNMT	PTAFR	ADH1C
KCNMA1	OPRM1	NAAA
CASP9	OPRK1	FABP3
SLC29A1	PSEN2	PRKCA
ADORA3	PSENEN	S1PR3
DNPH1	NCSTN	HAO1
ADORA1	APH1A	PPP1CC
SLC28A2	PSEN1	PPM1B
DNMT3B	APH1B	SLC22A6
CDA	PPP5C	PPARA
ADORA2A	PPP1CA	PHF8
AMD1	PPM1B	GSTK1
P2RY2	PPP1CC	PAOX
P2RY6	HSD11B1	ACP1
AHCY	SLC2A1	HMGCR
SRM	ADCY1	KDM2A
DTYMK	VDR	RARB
GAPDH	FOLH1	CDC25B
ADK	GLRA1	PTGER2
QARS	GLRA2	EPHX2
HSPA8	PPM1A	TBXAS1
P2RY4	PTGS2	CDC25A
HSPA5	PYGL	HSD17B3

TOP1	PYGM	PPARG
SMS	PYGB	TBXA2R
DOT1L	CA1	FOLH1
P2RY14	ACHE	PTGER4
RARS	PDE10A	PTGIR
KMT5C	CA9	PLA2G4A
SETDB1	SLC6A3	FFAR4
P2RX1	ADRA2A	GABBR1
TARS	ADRA2C	FABP4
TK1	ADRA2B	PTGFR
PRMT7	SLC6A4	PPARD
ADORA2B	CYP11B1	S1PR1
IMPDH1	GABRA2	TRPV1
FUT6	CYP11B2	CNR1
RNASE1	CA2	KDM5C
RNASE2	C1R	CPT2
HARS	KCNK2	PPM1A
RNASEL	PLG	PRKCE
GPR17	KLK1	VEGFA
HLCS	ELANE	TOP2A
P2RY11	C1S	G6PD
ADA	ADRA1A	CYP17A1
IARS	PSMB1	SERPINA6
PARG	CTSH	SHBG
EZH1	PIK3CD	RORA
SUV39H1	ADORA1	NPC1L1
DNMT1	CXCR2	NR1H3
RAC1	ADORA2A	GABRB2
IMPDH2	GABRB3	ESR2
MARS	MET	AR
P2RX4	HRH3	MGLL
ATIC	LIPE	SQLE
PRMT1	HRH4	FNTB
PLCG1	ALOX15	FNTA
UMPS	GRM5	ICMT
KMT2A	EGFR	PHLPP1
CDC42	PDGFRA	BCHE
TK2	P2RX7	AKT1
EHMT1	PLA2G7	GPBAR1
TPMT	FKBP1A	SRD5A2
CD69	CES1	ESR1
P2RY1	TRPM8	SRD5A1
SETD7	PIM1	SHH

HPRT1
ADH1A
PDCD4
NPEPPS
POLA1
PAX8
PNP
INMT
LGALS7
LGALS7B
EHMT2
MAP3K7
HSPA1A
LGALS9
POLB
MGAM
NEU2
NEU4
PLA2G4B
PLA2G2C
P2RY10
SLC25A20
LPAR3
PAM
LPAR4
GPR34
LPAR6
GSTM1
LPAR2
GGPS1
PLA2G5
FAAH
NOD1
EPHX1
FDPS
CES1
PLA2G2D
LPAR1
ADH1C
S1PR2
SPHK1
CDC25C
ADH1B

ADORA2B
TAAR1
HSP90AB1
TNKS2
CA12
PTPN1
LRRK2
TSPO
GABRB2
GABRG2
PDGFRB
PIK3R1
GABRA5
METAP1
TLR4
NAAA
KDR
MGLL
CES2
BCHE
NISCH
CTSS
PTPRC
CTSL
MAPK8
FABP4
PPARA
FABP3
FABP5
PPARD
FABP2
FFAR1
SLC22A6
CDC25A
AKR1B10
POLB
PHF8
UGT2B7
NR1H4
CYP19A1
SERPINA6
SHBG
HSD17B3

SREBF2
CYP19A1
NR1H4
UGT2B7
NR1H2
VDR
GC
CDC45
CD81
CYP24A1
PTPN2
PTPN1
FGF1
AKR1B10
PTPN6
HSD11B2
IL1B
PTPRF
HSD11B1
TAS2R31
ABCB1
CA2
CA1
RORC

NAAA	G6PD
LAP3	GABBR1
THRB	PTGER2
CA3	KDM2A
TLR2	KDM5C
PLA2G2A	GPBAR1
FABP3	NPC1L1
PRKCA	FNTA
THRA	AR
S1PR3	HAO1
HAO1	PTGFR
SLC22A8	GSTK1
PIN4	LTA4H
WRN	HSD11B2
PPP1CC	NR0B2
PPM1B	CDC45
PPID	PPARG
KAT2A	RXRA
CA14	CYP26A1
SLC22A6	CYP26B1
PPARA	RARG
BBOX1	RARB
OXER1	RARA
GNAI1	FAAH
GNAI3	TERT
CYP4F2	FABP1
GABRQ	FFAR4
KDM7A	CACNA2D1
DNM1	RXRG
COL4A3BP	ALOX12
GNAO1	PTGER4
ACER2	RXRB
MPEG1	SLC16A1
ADH7	ABCC1
KDM5A	EPHX2
POLM	CHRNA7
PHF8	PTGES
GSTK1	TBXA2R
GPR174	PLA2G4A
PAOX	SCD
BHMT	HTR2B
SLCO2A1	RORB
SELL	MAPK1

SLC6A11	RORA
SMPD2	SAE1
LTB4R	HNF4A
ACP1	FNTB
SELP	UBA2
ENPP2	VEGFA
HMGCR	FGF1
KDM2A	FGF2
PLA2G4C	HPSE
KDM4A	HSP90AA1
RARB	CDK1
CDC25B	LGALS4
KDM4C	LGALS3
PTGER2	LGALS8
EPHX2	DRD1
GSR	DRD2
POLH	ADRA1D
TBXAS1	HTR2A
ENPEP	HTR2C
CDC25A	DRD3
POLL	CYP2D6
POLK	HTR6
S1PR4	HTR1B
PLA2G10	RORC
HSD17B3	HEXA
S1PR5	HEXB
PPARG	OGA
TBXA2R	SLC6A2
FOLH1	STAT3
DAGLA	P4HTM
PTGER4	TRPV1
PTGIR	PAM
PLA2G4A	CPT1A
FFAR4	KCNH2
SLC22A1	GRIN1
GSTA1	ACACB
GABBR2	MTNR1A
GABBR1	MTNR1B
PTGER3	GRIN2B
FABP4	PDE4A
PTGFR	PDE4B
GPR35	NR3C1
ATG4B	TOP2A

KDM4E	CYP17A1
GABRR1	C5AR1
PPARD	SIGMAR1
S1PR1	PGR
TRPV1	SRD5A2
CNR1	ESR2
KDM5C	GC
CPT2	S1PR1
NAALAD2	F2R
EGLN3	KCNA5
PPM1A	PIM3
PGA5	HDAC6
PRKCE	NR1H3
VEGFA	TNF
FUT7	EPHX1
IL6ST	CA4
PLCG2	NR1I3
LPAR5	CNR2
APEX1	S1PR3
TOP2A	ICMT
GABBR2	CCR1
KDM4E	HTR1A
G6PD	FASN
CYP17A1	CNR1
SERPINA6	CTSK
SHBG	CTSB
GABRD	TACR1
RORA	SLC6A7
NPC1L1	P2RX3
NR1H3	CCR8
GABRB2	KCNJ11
SLC22A3	KIF11
ESR2	PGGT1B
AR	CCKBR
ASAH1	PANK3
MGLL	CDK2
SQLE	SCARB1
LSS	IMPDH2
FNTB	DRD4
FNTA	CHRM3
ICMT	CAPN1
FDFT1	NPY5R
BCL9	PAOX

TTPA	OXTR
PHLPP1	MAPK10
BCHE	AVPR2
AKT1	HTR7
GPBAR1	HDAC4
SRD5A2	HCRTR2
ST3GAL1	HCRTR1
ESR1	ABL1
SRD5A1	MAPK14
SHH	CPT2
SREBF2	SCN9A
EBP	CACNA1H
CYP19A1	AVPR1A
ABCB11	CHRM1
ABCC4	PTGER1
NR1H4	KCNJ5
EPHA7	KCNJ6
SLC10A1	TAOK1
EPHA5	TAOK3
EPHA2	F10
EPHA4	LIMK2
EPHA8	ECE1
EPHB3	DDAH1
UGT2B7	APLNR
NR1H2	PDE5A
VDR	SLC6A9
GC	SOAT1
CDC45	NR1H2
CD81	MME
CD4	KCNJ3
CYP24A1	HCAR2
PTPN2	F2
PTPN1	PRSS1
CYP27B1	CTRC
FGF1	SQLE
AKR1B10	BACE1
PTPN6	HSD17B1
HSD11B2	POLA1
IL1B	STS
F3	CASR
PTPRF	GSK3B
HSD11B1	NQO2
TAS2R31	CYP27A1

ABCB1	ADAMTS5
SLC10A2	CDK9
CA2	BRD4
CA1	BRD3
GBA2	RHOA
RORC	PRKCB
UGCG	NTRK1
EPHB1	RET
EPHA1	NR3C2
EPHA6	MPO
	HTR5A
	MAPK9
	CCND1
	KCNK3
	KCNK9
	CYP24A1
	TBXAS1
	MMP1
	MMP2
	CDK4
	GABRA3
	GABRA1
	AKT1
	PHLPP1
	ILK
	ESR1
	PTGS1
	ALOX5
	CCKAR
	ADK
	AURKB
	AURKA
	JAK3
	JAK1
	MCHR1
	PDK1
	BACE2
	CTSD
	CYP51A1
	SREBF2
	CYP2C19
	CHRM2
	DHCR7

PTPN6
CDC25B
NOS2
PREP
PTPN2
ATP12A
ADORA3
MAPK3
PTPN11
PTPRF
PLA2G1B
ACP1
SRD5A1
NR1I2
TRPA1
CCR5
ADH1A
TYMS
ADH1C
GLI2
GLI1
PDE4D
CD81
PARP1
PTGIR
HSD17B2
HRH1
CCR2
AKR1C2
AKR1C1
OPRD1
FLT3
HSD17B7
NAMPT
SCN5A
TEK
IL1B
JAK2
ROCK2
CHEK1
PTGDR2
MTTP
APOB

CXCR3
ABCG2
FPR2
GAA
MDM4
MAP3K11
MAP3K9
MDM2
TAS2R31
TNKS
SHH
