# Supplementary material for: Network Pharmacology-Based Study to Uncover Potential Pharmacological Mechanisms of Korean Thistle (Cirsium japonicum var. maackii (Maxim.) Matsum.) Flower against Cancer
Source: Molecules. 2021 Sep 29;26(19):5904. doi: 10.3390/molecules26195904 (PMC8513069; doi:10.3390/molecules26195904)
Supplement: Supplementary file 1 [file molecules-26-05904-s001.zip › Supplementary Table S2.pdf]

The number of 4,245 cancer-related targets from TTD and OMIM databases
EGFR
FGFR1
PDF
PLPRO
JAK1
JAK2
ACHE
AKR1B1
5LOX
CA1
CA2
CA4
HER2
ERBB4
ESR
FGFR2
FLT3
HDAC1
INSR
LCK
MMP1
MMP2
MAOA
PDE5A
PDGFRA
RET
SRC
BRAF
MTOR
TRKA
ABL
ATK
KIT
KDR
ALK
AR
CYP19A1
CB2
CA6
CA14
PRSS1
F2

F7
F10
CDK4
CDK6
AKR1C3
ESR2
FSHR
FYN
CCKBR
NR3C1
GCP2
GSK3A
HSP90A
IGF1R
MMP7
MC4R
MAOB
ABCB1
TRKC
OPRS1
ODC1
PIK3CA
PIK3CD
PIK3CG
PLG
PDGFRB
PARP1
COX1
PRKCG
MET
ROS1
RRM2
AMD1
SMO
S1PR1
CYP11B1
S17AH
STS
TK1
STYK
PLAU
VDR
HTR2A

ACACB
ADORA2A
ADRB2
AOX1
ALPL
APP
BCL2
ABCG2
CD22
CD19
TRKB
CXCR2
CXCR4
CB1
CD38
CTLA4
DHFR
TOP1
TOP2B
D2R
D3R
D4R
EPOR
FXR
FASN
ATP4A
NMDAR2B
GCSFR
IDO1
IFNG
IL4R
IL1B
CD20
NGFR
MR
CHRM1
MOP
ODF
PGR
PRKCA
F2R
NQO2
RXRG

SERT
STAT3
ATPA1
TACR1
CD4
TERT
TSPO
TNF
TYR
AXL
KCNH2
SCN5A
XDH
CD80
ADORA2B
ADRA1A
AT3
BDKRB2
TXNRD1
D5R
EDNRA
EDNRB
EEF2K
FGF2
FOLR1
GABBR
FDPS
CSF2RA
GNRHR
CSF3
CSF2
GHSR
GUCY2C
HB
IMPDH1
ICAM1
IFNGR2
IFNA2
IFNB1
IL12B
IL2
LEPR
LDLR

LHCGR
CD33
CHRNA7
NET
NFKB
IDH1
CELA1
PIGF
PDL1
PD1
PTGER2
COX2
SSTR1
SSTR2
SSTR3
SSTR5
CD3E
TSHR
TLR7
TGFB1
TRPV1
VEGFA
VEGFB
CACNA1G
CACNA1H
CACNA2D1
SCN10A
MEK1
MEK2
IMPDH2
EIF4A
BCRABL
ADRA1D
CTNNB1
DPYD
TOP2
GPR119
IL11RA
GNRH1
MRM2
SRD5A
SPTATPASE
CD28

PRDX5
TYMS
CGM1
UL30
TNFRSF8
MCL1
RIR
CS1
ANO
GCS
SLC19A1
ESRR
TUBB1
PLAUR
MAP
SLC46A1
ROCK1
SLC47A1
HDNA
HA
SAM
HDAC6
CYP3A4
CA9
FGFR3
GSK3B
HDAC2
MMP13
MMP9
AKT1
ADAM17
FLT1
VEGF
ACAT1
ADORA3
TEK
AURKB
AURKC
CSNK2A1
CTSK
CHK1
CCKAR
CDK1

CDK2
CDK9
EPHB4
ERK2
HDAC4
MMP12
MAPK3
SIRT1
NOS2
PLK1
PREP
NQO1
AKT3
RARA
S6K1
P38A
TGFBR1
MDM2
FLT4
SCN9A
WEE1
YES
ANGPT1
ANGPT2
BIRC5
BCLW
BCLXL
ATM
CCR2
CASP3
CTSL
CTSS
CD40
TP53
CDK7
DNMT1
DNMT3B
PRKDC
DUSP1
SERPINE1
EZH2
EPHB2
ERBB3

FGFR4
FN1
FAK
GCR
HGF
ITGAV
ITGB1
IL2RB
IL18
KIF11
LSD
MPIP2
RON
ADAM10
MME
P2RY2
PIK3CB
FAP
PTGER4
KLK3
PRKCB
PTP1B
ROCK2
PIN1
RAD53
PIM1
SGK1
SPHK1
TLR3
TLR4
TRAILR1
TRAILR2
TGFB2
JTK8
XIAP
ACVRL1
APEX1
LPA
FAS
BSG
BRDT
BRD2
BRD3

BRD4
S100A9
CD66E
CASP9
BIRC2
BIRC3
CNTF
CLUSTERIN
MPS1
UBE2T
EFGR
HSP90B1
EPHA2
EPHA3
XPO1
ERK1
FTASE
CCND1
GAST
GRPR
GLIPR1
GART
NRAS
HIF1A
IL13RA2
IL1A
IL10
IL21
LTF
LTB4R
LTBR
CSF1R
MKNK2
MKNK1
MAPKAPK2
MAGEA1
STEAP1
MYC
NGF
NRF2
CD134
SELP
P2RX7

P2RY6
PAK4
PI4KA
PDPK1
HBEGF
RAF
PPP2CA
FRP1
SSTR4
SPHK2
OX40
TGFBR2
CD40LG
TNFSF10
TLR2
TLR9
TRPV6
TNFR1
VWF
WT1
OGDH
CD276
BS
CD27L
SLC44A4
CGB
IGF2R
CD55
CTGF
P105
DUT
ELF4EBP
EGFR8
EGFL7
GS
HSP70
HSP90B
EPAS1
ITGA2
ITGA5
ITGB3
IFNAR1
IL15RA

IL23R
MELK
MSLN
MUC1
MYCBP
ROR1
NOTCH2
NOTCH3
NCL
MDM4
CDH3
PRLR
ACPP
RNF43
SEMA4D
CD27
TNC
TXN
F3
TLR5
TLR6
TF
DDX5
MSMB
MAGEC1
GPA33
CLDN18
DLL4
DKK3
ENG
CD44
FACT
FUT3
GGTASE1
GLS
GPC3
HSP27
HIF2A
CD37
CD47
LOXL2
MAGEA3
MAGEA4

MAGEC2
L1CAM
NKG2A
RORG
NPM1
TPBG
MUC16
PDDX5
PDGFA
PTEFB
PORCN
RSPO3
SLC5A5
SLC39A6
SPTBN1
CD3G
TFF1
TUSC2
EPCAM
NEDD8
GLA
NYESO1
DLL3
CD248
IGF1
IGF2
JNK
LAG3
KISS1
MKRAS
KLRK1
OGFR
PAKT
PD2
PROM1
CRBN
TMEM97
SLITRK6
CD8
MTORC2
ACT
ARG
DPOL

RNAP2
FGFR3-TACC3
GNEF
HIAP
MTORC1
NAE
PSCA
SOAT
GD2
MIR34
FRD
HDNAMIN
PG
HDAC8
CASP8
CTSB
CTSF
HDAC10
CTSV
ENPP2
PDHK1
PAR2
BCRABL T315I
P450
HSPB1
KISS1R
ABCB4
MAN2A1
HRAS
ACKR3
CYP46A1
P2RY11
P2RY4
MSI2
PLA
FNTB
MAP3K8
JNK2
MPIP1
MPIP3
NCOA3
PRKCZ
ACK1

BMPR2
HPH2
IKKA
ILK
PLK2
PRKCI
RHO
ATAD2
CCNA2
CCNE1
CARM1
KRAS
LGR5
KDM5B
PBK
ATIC
PRKD1
RACK1
RCVRN
S100A4
BRCA2
GCLM
IL13RA1
MLTK
COT
S1PR3
CTCFL
DDEFL1
SNCG
CX50
RHBDF2
MPHOSPH1
LAMC2
SGO1
BAGE
MAGEA2
NGEP
PAGE5
LSM1
MIR191
IL2R
NAC
PDE1

5HTR
ADRA2
CA
DNMT
HDAC
IFNA
MAP3K
PDGFR
CAC
MEK
MDR
CHRM
NMDAR
OPR
RAR
PTK
VEFGR
HYAL
MAO
PARP
ROCK
SP
V1R
NAV
ACH
AMCG
CIC
EMBP
HS
NKA
PAM
PTGR
TAG72
KCNQ
BET
CASP
NCAM
PRKC
S6K
P38
TNKS
CTR
CDK

TOP
DR
FGFR
HLAA02/AFP
IFNR
MMP
PLC
PK
RXR
PP2A
TLR
NGCGM3
AHR
CSK
CSNK2
CC
CRF
FGF
FKBP
GALT
ICH
JAK
STAT
LYA
NA
PCC
REC
RNASE
STK
ST
SBC
TCR
THR
TUBP
WNT
CDE
MGT
PGP
PMH
POL
ADCY
TMET
FAPP

PPTF
CA12
TNFSF13B
IL12
FGF23
AURKA
HAMP
LTA
TNFRSF12A
PTK6
APRIL
TNFRSF18
TNFRSF13C
CD137
PKM
TSG101
TWEAK
TNFRSF17
TNFRSF13B
VHL
TPT1
ST14
LATS2
ERAP1
FASLG
KLK8
NM23H1
RANK
TNFAIP3
CD178
CD258
CD270
IL32
LTB
TNFSF15
RHCG
PLXDC1
TRIM59
TNFR2
TP53BP1
PINX1
FAT1
PRAME

TNOX
TNFA
TUSC
KH1
TAP
IFNAR2
PTGFR
LTB4R2
ROR2
IRAK4
EFGR8
HSP9OB
ENPP3
PFKFB3
PTK7
TACC3
KDM4C
LRP
LR
DDR1
LDHA
UBE3A
TPX2
NEK1
NEDD9
LDH
RBPJ
SCCA1
TORC1
HCAP
CD24
MG7
HLAA2
AFP
S1PR
SSTR
HDGC
TOC
BROVCA1
CRCS1
BROVCA2
BROVCA3
SLC45A3

PRNCR1
LUCAT1
CRC
HLRCC
NMTC1
HMPS1
MMRCS1
HNPCC2
CRCS3
CRCS10
ODCRCS
HNPCC6
FCTCS
CRCS12
HPCX1
NMTC3
HPC8
HPC7
CRCS2
CRCS5
CRCS6
CRCS7
CRCS8
CRCS9
CRCS11
CT45A2
CT45A3
CT45A4
CT45A5
CT45A6
CTAG1B
CT45A1
BRINP1
CTAG3
BLCAP
SDCCAG8
BLACAT1
SCHLAP1
PCAT2
CASC8
CASC21
CASC19
CASC11

CCAT1
ALDH2
CHEK2
NMTC2
PAIS
HPC1
HPC9
HPC12
HPC13
SQTL3
HNPCC8
BROVCA4
PNCA4
HNPCC4
HNPCC5
HNPCC7
HPC2
NMTC4
NMTC5
SSPCS
CAPOK
PNCA5
MMRCS2
MMRCS3
MMRCS4
HPCX2
OVCAS1
LNCR1
HPC5
HPC10
HPC11
HPC14
HPC15
LNCR3
LNCR4
LNCR5
BRCA1
DCC
STEAP2
ANO7
TFDP3
CT47A1
CT47A2

CT47A3
CT47A4
CT47A5
CT47A6
CT47A7
CT47A8
CT47A9
CT47A10
CT47B1
EEF1A1
MCC
MYCL
FGF4
LCO
SEMG1
PASD1
FUNDC2
CD82
PTPRJ
DAB2
TUSC3
GPR68
PTEN
ARMCX1
ARMCX2
ARMCX3
CTAG2
LDOC1
CT47A11
CTAG1A
STARD8
DPH1
SLC43A1
HIC1
ITGA9
DLEC1
SYT7
DLC1
INTS6
AKAP13
BCAR3
01-Dec
PCA3

NCOA6
PBOV1
CADM1
HID1
MBOAT7
BRMS1
CASC3
DCD
CDK2AP1
VWA5A
BCAR1
BCAS1
RNF139
ORAOV1
NDC80
RHOBTB2
CCAR2
TCIM
HIC2
IGF2BP3
NEMF
BLID
AKIP1
AGR3
FAM84B
ARID4B
PCAT4
STARD13
TBC1D3B
PDZD2
ANKRD30A
AGPAT9
CENPW
HEPN1
TMPRSS11A
ECRG4
USHBP1
VOPP1
GAEC1
MSMP
HULC
MED19
MACC1

CASC2
GLDN
ALKBH3
CHD1L
LRRC26
BCAR4
KCMF1
COLCA1
COLCA2
PCAT1
FALEC
GACAT2
GACAT3
SLC38A9
NBAT1
C4ORF46
PCAT29
FAM168A
ANKRD30B
ASAP3
CASC15
MYCT1
TMEM8B
CASC1
ERGIC3
CWC27
SPINK7
UCA1
PCAT18
LINC01488
COX6B2
PCAT19
VWA2
ENTR1
KIAA1211
LINC00958
BRMS1L
CCDC33
LETMD1
MLH1
CDH1
MSH2
APC

CDKN2A
LFS
LFL
AT
ATA
KRAS1P
PMS2
MSH6
PALB2
ESR1
TGCT
ERBB2
FAP1
RCC
NRC1
RAD51C
MLH3
ELAC2
CMM1
GLM1
GLM
CWS1
LDD
SMAD4
FH
FH1
STK11
MSR1
RAD51
RB1
PJS
CD274
MRTES
PPARG
MUTYH
NFKB1
FHIT
FRA3B
MTORC
HOXB13
RAD51D
KGFR
BUB1

SPOP
FANCS
FANCA
RASSF1
FBXW7
FOXA1
PARK2
NBN
BAP1
PKM1
CDKN1B
HABP2
PTGS2
AXIN2
DAB2IP
NBLST1
RUNX1
SOX4
SEC23B
VHLS
BWS
BWCR
GSTM1
KMT2A
ERG
POLD1
RNASEL
BARD1
SFRP1
BRIP1
YAP1
FAP3
WWOX
FRA16D
KLF6
TNFSF11
SRD5A2
ARL11
CLL
XPA
BLM
CWS7
PPM1D

LCN2
RUNX3
CDH13
SMAD2
TCF7L2
SDHD
XBP1
MIR21
JPS
ADH1B
NAT1
RBM5
PI5
MXI1
DICER1
POT1
TMPRSS2
FLCN
MIR34A
MEN2A
WRN
PHB
NOTCH1
BAX
SHH
TNFRSF11A
CLDN1
MIRLET7A1
EP300
XPC
RABL3
CYP2A6
CHL
CML
ES
PNE
TPDS
CYP1A1
PLA2G2A
EWSR1
PTHLH
POLE
LZTS1

SLC5A8
TOX3
MIR126
MIR126*
CHH
ETV1
BCR
MC1R
SEMA3B
ING1
MTA1
LMO4
CDK8
TP63
FANCM
MIR16-1
GALNT12
BHD
MESOM
PHGDH
MTAP
TUBB
ID1
GPC1
HMGA2
MSH3
CAV1
ATR
HYAL2
SNAI1
MUS81
NRP1
BUB1B
ARID1A
PALLD
CYP4Z1
HNSCC
FANCN
H19
CDKN1A
CHRNA5
HSP90AA1
LGALS3

GALIG
XRCC2
BAG4
MED1
RECQL3
RECQL2
RB1CC1
KMT2D
SMARCA4
STARD3
SNORD50A
NF1
PGL1
AML
IBD1
XPF
XPF/CS
HFE1
CHRNA3
GHRH
PTN
BMI1
CEBPB
ABL1
STAG2
OPCML
SMARCB1
MIR221
MIR222
TARBP2
DIRAS3
ZBTB7A
PER1
DNMT3A
CLN3
NF2
POSTN
NAT2
TRIM62
RCCP1
FERLK
RTPS1
SPS

PERM
ST3
ALL
CYP4Z2P
ZFHX3
FGF8
NFE2L2
EIF4G1
GPX4
INHA
IDH2
NME1
RAF1
SDHB
APOBEC1
PDCD1
FEN1
ETV6
XRCC3
TERF1
SFN
CSE1L
CASP10
CASP10B
BEX2
MBD4
TNFRSF10B
RAD54L
CTCF
CHFR
NOL3
ARC
BBC3
GNMT
PKHD1
TERC
ROBO1
DUTT1
SLC22A18
FOXO3A
LASP1
PPP2R1B
MTHFR

APOBEC3B
MIR27B
OR51E1
DEPDC1
MIR200A
ATG2B
HMGCR
LDLCQ3
BCNS
EPPK
MDB
PCTT
MVA1
FANCD1
GLM9
PML
SLPI
CTNNA1
NR4A3
GRN
IL6
LRE1
PTPN11
ANXA7
ZEB1
MTCYB
MTCO1
SYK
STAT1
CXCL12
ACVR1B
HIP1
VSIG1
BEX1
RECQL4
PRMT5
IL24
RAD54B
BECN1
MERTK
ABCC5
PCGEM1
KLK9

TMEFF2
EIF5A2
NKX3-1
CHD1
BLMH
MAD1L1
SMAD7
SMAD3
SMARCA3
MPO
NPRL2
S100A14
SMYD3
FBXO31
CYP17A1
CIP2A
OR51E2
RPRM
ZKSCAN3
TRIM68
VTRNA2-1
MIR31HG
ARFGEF3
RNF208
SCS
ACNINV1
UL
MTC
MEN2B
RTS2
TDH3
CF
EV1
PARK8
FAP2
LFS2
BCC3
BCL6
CEBPA
CBFB
CYP2D6
TRIM24
RBM6

ERCC2
CD34
IRF1
LOX
IL12A
ETS1
PTPRF
ARID4A
POLR2A
MYB
THRA
THRA2
THRB
TNFRSF1A
TMSB15B
EMX2
LGALS3BP
DAPK1
MAP3K1
BIN1
VEGFC
CYP1B1
STK26
BCL10
RGS6
ERN1
SOCS3
ATG5
PDGFRL
TCF7L1
TSC1
FGF20
PI14
AGO2
PTP4A3
TMPRSS4
FRK
NEO1
TP73
SPRY1
HIST1H4A
SAFB
ZNF217

PTPN14
ABCC6
SEZ6L
CREB3L4
CPA4
DOCK4
SASH1
TNS4
CABLES1
NECTIN4
ST6GALNAC5
ST6GALNAC1
MTDH
MIR29B1
HOTAIR
MIR200C
ATG9B
STING1
RFX6
ING4
PIM3
NOP16
MIR449A
PRDM5
ELOVL7
RNF126
C1QTNF9BAS1
CAVIN3
RALGAPA2
AL
JPHT
IPF
HRZ
THCYT1
IMF1
M7MLS1
RTPS2
ACTFS
BMFS6
TGCT1
B4GALNT2
CEACAM5
CDA

PIK3R1
JUP
TNFRSF25
GDF2
PATE
MAGED4
F9
EGR1
SELE
GAL
FUS
GNAT1
HPN
MGMT
MUC6
PVT1
CBL
ODCP
PTPN3
PRKCE
RPA1
PCDH11Y
TFPI2
PTPN12
PMS1
PTPN13
GABPA
SLC16A1
NUP98
SEMA3F
MAP2K4
KAT6A
FOXP3
PDLIM4
MBD2
RPS20
KLK4
POLH
PNMA2
RBBP8
SLC38A3
GADD45G
BIRC6

KEAP1
EXO1
SUZ12
XAF1
PYCARD
PLCD1
PTPRH
EIF4A1
DUSP6
ADGRL2
CYB561D2
ZMYND10
CACNA2D2
EML4
KRT9
NFKBIZ
SOX9
EXT1
IGF2BP1
ABTB1
LRP1B
FRA10AC1
FRA10A
RAET1E
MIR155
BIC
ERCC6
PGBD3
MDGA1
EHBP1
MIR125B2
MIR29A
MIR29C
MEX3C
ZC3H12D
GSDMA
SLC22A17
NOL8
SART3
PRDM14
PGAP3
MIEN1
RIOX1

KLLN
MUC13
MIRN101-1
HJURP
EMSY
TET2
MIR93
NCEH1
UQCRH
FAM72A
TSPYL5
MIR495
MIR1260B
CAHM
TNFAIP8L3
CUZD1
GPATCH2
SH3YL1
STARD10
PTCSC1
DDIAS
GKN2
LASTR
CCL2
DESMD
CMM2
POROK1
XLI
RMS2
XPD
AIS
GIST
DFSP
XFEPS
A1ATD
NPCA2
GLM5
APEH
APOB
ABO
CDC25A
LIG4
IQGAP1

TRIM37
AIFM2
UTRN
EDN1
EIF4E
GSTP1
GSTPP
FUCA2
GPT
HOXA5
HOXA9
IGFBP2
IGFBP5
IL1RN
ALOX12
LSP1
CXCL1
NME2
MITF
CEACAM6
LEP
MYCN
PAX2
SPINK1
SULT1A1
PTPRG
SFTPA2
RPN2
CCL5
THY1
TFDP1
TFE3
NNMT
CREBBP
NUDT1
PCGF2
CHUK
IHH
TGFBR3
RTN1
PLXNB1
SIX1
LIMK1

H2AFX
MAGEA6
PAGE4
PAGE1
XAGE1D
INGX
GAGE1
GAGE7
DAB1
BUB3
PNMA1
GNG7
CBLB
DYRK1B
MUC12
ACTN4
FRAT2
PLK4
PDS5B
ZNF350
AGAP2
HOXC10
SCGB2A2
IL23A
EBAG9
PIGN
RNF26
KRT23
AGR2
CYP27A1
TNS3
OGG1
NR1H3
MAP3K7
NTHL1
NFATC4
IFRD2
RBBP9
RAD51B
PLAGL1
S100A11
IL17A
NAA80

KMT5A
CDC73
BCAS3
BCAS4
STK33
MALAT1
MIR23A
UNC93A
RRAGD
FAM107A
UBR5
PTPRT
RNASEN
CHD7
CARD8
MIR15A
ST6GALNAC2
AGK
LZTS2
PFKM
HSPH1
MIR23B
HACE1
NRG4
SMG5
STEAP4
KLHL7
ARMC3
HMGN2P46
MINDY3
WDR77
MIR335
ABCB5
MIR141
MIR25
GOLPH3
TUSC5
B4GALNT3
ERGIC2
FAM120A
RNF11
PYHIN1
PIGU

RAB25
MIR106B
MIR100
FEZF1
MIR661
TMEM184C
CGAS
POLR3A
G0S2
MIR616
MIR99A
MIR570
NUPR1
ZNF516
RHBDD2
MIR149
MIR149*
TMEM79
MMS22L
MIR301A
RNF180
PPM1H
SUSD3
PHF21B
TMEM9
TMEM45A
C8ORF17
TMEM243
ZNF703
ZNF668
DEUP1
SOCS2AS1
PARM1
UBE2F
CEACAM21
DUSP23
TEKT5
SLC39A9
IRS1
LEF1
DMSMFH
BL
XLA

MEN1
MNG1
HRPT2
MCD
DKCX
HHS
XLP1
PPB
CPP
CPC
PV
SHEP2
TYRSN1
XPG
XPG/CS
FMRD
MEN4
CMM9
CMM10
DKCA6
DKCB7
FAP4
EDSPD2
EV2
DIJOS
HSR
WM1
FHL1
BCC1
ASCL1
ACY1
ACLY
CALR
NUP214
ATF1
VIM
PFKP
ENO1
CDKN2C
SPAG1
MVP
SPINT2
GADD45GIP1

RRAGB
XAGE1B
TOP2A
EGR2
ENO2
FABP3
FOXO1A
GLUD1
SHMT2
HSPA1A
HSPA2
HOXA7
HOXA10
HOXA11
HOXB9
ITPR3
WNT2
LIF
AKT2
ETS2
YES1
WNT5A
SPP1
REG3A
SPARC
TPMT
E2F1
TSC2
UBE7
PRAF2
PSMD10
FAM46D
YY1
CDX2
AQP5
S100P
NKX2-1
HMGA1
HMGA1B
ETV4
FOLH1
HMMR
BCL2A1

ABCC2
YWHAZ
SKP2
GGH
EVPL
NTN1
HAS2
TMSB4X
SSX4
ZBTB33
FGD1
GAGE12I
SPANXB1
BUD31
BTRC
MADD
SOCS1
AXIN1
BCL2L11
BAG3
HYAL3
B3GALT5
MAD2L2
ARPC1A
ARPC1B
PPP1R1B
TM4SF5
HPSE
ARHGEF12
CTNNAL1
TBK1
GDF15
HDAC5
WFDC1
CYP3A7
ADAMTS9
KLK13
SF3B1
BIRC7
BCAS2
RALBP1
STRAP
GAB2

SIRT7
CAPN9
GKN1
DIRC1
AXUD1
SCGB3A1
AHRR
SRGAP1
NLRP1
FTCD
TIMP4
ARMET
THRSP
NCOA4
TERF2
SH3BP2
HTRA1
WWP1
KLK6
MNAT1
HIST1H3A
NR1I2
CUL4A
ATF3
IKBKB
ERBIN
IHPK2
HYAL1
TGIF2
POTED
C15ORF48
MTSS1
PSMC3IP
MIR17
MIR17*
HAL
PDLIM2
PHF19
KDM4B
PRAP1
SULF1
LZIC
PRR13

WASHC5
PDRG1
SLC43A2
TMEM189
NT5DC3
FAM84A
BNIPL
OCA2
STYK1
SRD5A3
BCCIP
S100PBP
CCDC136
MIR373
MIRN200B
MIR143
PREX2
MIR31
RRAGA
MRC2
RAB21
ARRDC3
CCAR1
RASSF6
SPA17
KLK15
SMARCAD1
RAB6C
PDS5A
LAPTM4B
ALKBH8
DSCR8
PTENP1
MIR148A
RHNO1
CUEDC2
MIR99B
MIR489
SCUBE3
MIR193B
DENND2D
DNAJC15
LSINCT5

SUSD2
CREB3L1
ZNF695
DRAIC
GPAT2
NRBF2
MFAP3L
MORC2
RPL34
MARVELD1
ARHGEF26
ST7L
SRMS
ARLNC1
MINAR1
ATP5PD
C2ORF68
CKAP4
SMYD5
PPARA
MN
PGL4
MMM
PWS
PWCR
PCS
DCR
RCCX1
WT5
TTD1
XPV
BOCA
CELIAC1
CHDM
CSA
CSTLO
CMEMS
FANCD2
HYDM1
GSD1A
ICF1
ANXD1
FANCI

XPB
LGSS
WABS
CMM8
BCC7
PFBMFT1
DBA13
THC5
BMFS3
SEMDK
PGL6
LE
DPP4
VCAN
CKB
CCND3
DEK
NFATC1
CLOCK
NAE1
DHX15
PLAAT4
TAB2
LPAR3
PSME3
WFDC5
ANP32C
MIR224
TCEAL7
DDIT3
LIG1
EPO
ERCC3
ERCC4
ERCC5
FPR1
CSF3R
RASA1
GHRHR
GNAO1
HPR
HSF1
HOXC6

SOD1
ITGB4
IFNA17
RPSA
LIPE
SAI1
ADAM11
RMRP
CCL7
MUC2
AFDN
NOP2
POU5F1
REL
JUN
AP1
PAX5
TAP1
PTPN1
SERPINE2
RPS15
RNU1A
SOX2
CLU
THBS1
TG
PRKAR1A
HNF1B
UGT1A1
UGT1A
MIR502
MIR718
HDX
MTND1
MC1DM3
MTND4L
MTCO2
MHC2TA
MATK
BCL2L1
HNRNPA2B1
HNRPA2
SEC13

MICA
TLE1
SS18
CCR7
MAPK14
CSBP2
PCM1
DDX6
TYRO3
AMPH
TRIM25
PTPRO
MCM7
EPHB1
TBX2
DYRK1A
CDKN1C
SIM2
NPRL3
PRDM2
SEM1
BMPR1A
PPP3R1
PTCH1
MYBL2
PEG3
ZIM2
ETV5
TFAP2C
TWIST1
CASP7
ATRX
FOXO4
DCX
DKC1
KDM6A
MED12
ENOX2
SPANXC
SAGE1
ZNF185
XAGE2
SPANXA2

UTP14A
SSX6
TSPYL2
GAGE2C
AMER1
FAAH2
MIR424
MIR322
PER2
CDK10
ASS1
WNT4
UNC5C
RPL5
NUMB
DACH1
SLC16A4
RFX4
RAD50
TPD52
KCNQ1OT1
GPRC5A
SLC25A11
RPL15
SCO2
ABCC3
TRPS1
POLQ
PI13
WNT6
PART1
IKBKE
DIABLO
ABCC4
TACC2
EPB41L3
BNIP3L
ITGB3BP
FOXP1
SMURF2
KLK12
FSTL1
MIR198

KLK5
FBXL2
BACE2
SCRT1
ANGPTL4
BASP1
NDOR1
TES
BHLHE41
ASPSCR1
JAZF1
SELENOF
GLS2
FXYD5
TRPM8
TTC4
ST13
JAG1
PAWR
NCOA2
EREG
SIPA1
APAF1
KLF4
GAL3ST1
FOXM1
CYR61
FOXC2
GPC5
ARNTL
KLK10
BACH1
NSD2
PCDH7
IKZF1
CUL2
CUL3
RAD17
UBE1C
LSAMP
AMFR
ZNF35
COQ8A

RASSF3
RASSF5
RAPL
SLC39A4
APOBEC3A
SAV1
CARD11
MYO18B
APCDD1
RPL39L
RNF20
DDIT4
UNC5A
UNC5B
KCNRG
HECA
GNL3
SAFB2
RFWD2
PPHLN1
VTCN1
SNIP1
DIRC3
HSPBAP1
RNF34
PINK1
IGSF11
LIMA1
DNAJA3
CSMD1
LACTB
AGGF1
CYGB
NAMPT
OSR1
PNPLA2
FBXO45
KNL1
IQCB1
SEH1L
SH3GLB1
KDM3B
LUC7L3

RASSF2
ARID2
MGLL
DUS2L
XRRA1
TBC1D2
CASZ1
MIR125B1
TTC9
ADRM1
NACC1
TTC12
SLC35B3
LMTK2
EPC1
LIN28A
LIN28B
MIR9-3
MIRLET7B
KIF14
CRNN
C3ORF35
NOL7
NALCN
NAPRT
MIR10B
DHRS4
PLA2G3
RNF8
ERF
KDM8
RPS27L
SAPCD2
MIR22
MIRN429
GLCE
GGNBP2
ICK
ABCF2
PDCD4
STRADA
WWC1
RSPO2

BOP1
PISD
BCAM
RNASET2
FILIP1L
MIR205
KMT5C
SAE1
UBA2
CLEC3A
EIF2AK1
PLAAT2
DEPDC5
PSD3
MIR1258
HIF1AAS2
MIR520H
STRN3
SIK3
C1ORF109
NPTNIT1
MIR7-1
B3GNT8
MIOS
MIR650
MIR574
MIR574-3p
RGS22
POU5F1B
GPX7
MIR190A
DEPDC1B
CDK15
NWD1
FRMD5
ARMT1
PARTICL
DCUN1D5
SRPRB
GSE1
PTCHD4
GSDMD
FKBPL

ST18
SCAF1
WFDC2
USF3
C10ORF99
ZNF664
STRIP2
EEF1AKNMT
RCCD1
ZNF768
NXPE4
SEC11A
NHLRC2
PRR15
BRD9
HECTD3
C19ORF48
ANKRD18B
CYP1A2
PITA1
PAP
DBA1
T2D
DUH1
HRPT1
CSCF
PD
MAS
PFD
CPPB1
PTRPLS
PKU
ECYT2
PRLMNS
WND
BRSS
ASPS
ADCC
HFS
NBS
BBS
GDMN
JMML

CMML
IMD54
B12QTL1
NBLST3
FANCO
CMM6
MDS
MBLD
PFBMFT2
CWS4
MSPC
CPPB2
DUH3
ALL3
POF8
MPLPF
HRPT4
CHTD5
LPFS3
EV3
RTS1
BPH
IH
SXGQTL1
EBVS1
HPV18I2
GBY
ST12
PPBL
GNRHR2
SERPINC1
IFNGR1
TRIM21
BCAT1
CALCA
CGRP
CDC25B
MORF4
CHRNB4
MMP8
CRYAB
CREB1
CDK3

VCAM1
AVP
NPII
TCF12
SERPINB3
SERPINB4
GPER
PFKL
PGAM1
WISP1
EPR1
RABIF
SOCS2
PXDN
ELF5
EI24
TP53I3
PTGES
GAS8
GAS8AS1
ANP32D
GOLGA5
XAGE1A
XAGE1C
XAGE1E
RPS14
FURIN
GGT5
SLC2A1
NR4A1
HOXD10
CD9
ITIH2
ITIH3
IGFBP3
IGFBP4
ITIH1
CXCL10
CASP1
IL4
TTIM1
CD5
MAX

MTR
CDC25C
ABCC1
MCL1L
NFE2L1
RELA
HNRNPA1
FGF3
FGF5
GLI1
NBR1
OXT
PAX7
CD36
PIP
MKI67
ZBTB16
EIF2AK2
MAP2K1
PPP1CA
PYCR1
RALA
EPHA1
PRCC
RBL2
SHBG
TFF2
TLX1
CD8A
LEU2
FES
TDO2
BLK
U2AF1
TMSB15A
BRAFP1
ZMAT1
MIR532
CDR1
FMR1
FRAXA
KDM5C
UTY

TSPY1
MXD1
ABCA1
MAP3K11
RRAS2
KCNMA1
MST1R
SLC7A5
DUSP3
RUNX2
FOXA2
RAPGEF1
ID2
PPARD
E2F2
E2F3
CDKN2B
AKR1C2
GPR4
ITIH4
CLCN2
TFF3
TCTA
PTBP1
LPP
HNRNPK
TFEB
MMP14
ST7
ARHGEF5
DHPS
CAV2
PGF
HK2
CKR2A
MIA
AFF3
FRA2A
MAD2L1
MAT2A
GRB14
PPP2R5C
GTF2I

EFNB1
TEX28
VBP1
MAGEA12
USP26
NXF2
SSX3
SSX5
MAGEA5
MAGEA8
MAGEA9
MAGEA10
MAGEA11
SH3KBP1
BCAP31
MAGEB5
MAGEB6
MAGEC3
MAGED2
WDR45
BRWD3
CXCR3
SPACA5
GAGE3
GAGE4
GAGE5
GAGE6
LUZP4
GAGE8
PNCK
PEA15
LATS1
GALNT4
TNFSF4
TNFRSF10A
RPS5
RPS9
RPS10
RPS29
RPL21
RPL27A
RPL28
RPS16

RAD9A
PHOX2B
CBFA2T3
GPHN
HELLS
SCD
ATF4
PTTG1
CXCL14
RNF6
USP6
HPV6AI1
SIRT2
TRIP10
TRIP12
PPARGC1A
LIMD1
ERG28
FZD4
PEBP1
LTBP4
LTBP4S
RRM2B
USP25
SLC39A1
COPS5
SRP68
RPH3AL
CYLD
UBQLN1
YWHAE
RECK
POLI
AICDA
TACC1
APPBP2
DHH
ASPM
PRDM16
HTATIP2
MEG3
CPNE7
ULBP1

HHAT
COX7A2L
ZEB2
RIPK3
TMC6
KCNK9
LAMP3
DSE
NOD1
CPSF3
ZNF331
PBRM1
LGALS8
NUDCD1
FZD3
TP53INP1
SIRT6
WNK2
WNT10A
ANTXR1
EGLN1
UGT1A10
PTPN23
DAAM2
CEACAM19
TPO
ADGRA2
PLXDC2
DMBT1
SATB1
NOVA1
SIAH1
ADAM8
MMP17
NR1D2
TGFB1I1
GML
NR1D1
PPIE
MICB
PTPNS1
TRAF4
PXN

USP7
PTPRK
LIMS1
FUT8
PAK1
FOXE1
CTBP2
TSPAN4
SEMA3C
ADGRB1
FSCN1
SCARA3
SPG7
PLK3
RCAN1
EEF1A2
IER3
E4F1
PLAG1
SMAD5
ENTPD5
KIF22
SLC27A2
BMPR1B
CST7
CHD4
TCF21
XRCC5
ZNF71
GLDC
TNK2
SUFU
ABCC11
ABCC12
ERC1
TNKS2
ST3GAL6
ZMIZ1
TSGA10
RGS8
PUM2
FILIP1
WWTR1

ING3
KLF12
PIBF1
DIS3
RDH11
YY1AP1
LIMS2
OSGIN1
UHRF1
HSPB8
TSKU
COA4
FAM162A
P2RXL1
WDR19
GULP1
CPS1
EMCN
ACRBP
DPYSL5
MMP21
MUC17
MRAS
PCBP4
ZMYND11
BRAM1
RAB3IP
SSX2IP
DCBLD2
GIPC3
SPECC1
GSDME
L3MBTL1
ZFYVE16
MTA3
KDM1A
HCAR2
CLDN23
WIPI2
WDR45B
ODR4
ACD
DND1

ARHGAP8
PIKFYVE
MIR17HG
MIR18A
NDUFA13
PELP1
NLK
LYPD3
MAP3K2
RSPO1
KLF17
ATG12
DDX58
NDUFAF2
NLRP7
ITIH5
REG4
CEP55
COL23A1
ST6GALNAC6
CEP290
PTOV1
MED25
CRIPAK
SLIRP
EDEM3
MTFP1
ORAI1
MED28
MACROD1
LEMD1
PKN3
WAPL
TIGAR
GMPR2
PRAC2
IL17RC
SOX17
MIR133B
SMG7
PPP1R15A
MIR197
CLDN12

MIRLET7E
KIF12
ADNP
PRICKLE4
NPL
POLD3
CIZ1
YBX2
FAM57A
DDX54
C1ORF76
CDCP1
NDUFAF4
MIR145
NLRX1
MINA
ZNRF3
MIRNLET7G
TNFAIP8
USP22
SERTAD3
14-Sep
EPB41L4A
SLC39A2
SLC39A3
CLPTM1L
TCHP
HBXAP
ING5
PPIAL4E
PHF17
TUSC1
MIR146B
MARVELD2
CPEB4
MIR187
ATP2C2
NRIP3
MIR449B
CDKN2BAS
KCTD3
TMEM127
PARPBP

SLC36A4
ZNF652
ARGLU1
MCIDAS
MARVELD3
MIR519D
MIR137
VTI1A
DHX33
AFAP1L1
SERINC2
FAM32A
PAQR3
MIR302B
CARMIL3
PTCSC3
ODAM
C1QTNF4
MIR298
TRIAP1
ATPIF1
BATF2
CAMKK2
MIR139
MIR139-3p
EPG5
LY6K
MIR551A
MIR30C1
MISP
B3GNT7
CERS6
SFMBT2
TLNRD1
NISCH
MIR185
MIR185*
CRNDE
NLRC3
C11ORF95
ZMYND8
SMOX
PRR11

WSPAR
SORBS2
TP53TG1
ADGRL4
SPAG17
ZBTB1
TBC1D16
A4GNT
SUSD6
SLC46A3
CLUAP1
CEP68
TRIM40
ENPP7
SEMA4B
LINC01194
LINC00663
C17ORF49
TP53TG5
SHKBP1
RPL31
UBE2Q1
TP53TG3
ZNF318
OGDHL
RALGPS2
TRIT1
DDX46
STRIP1
ZDHHC20
NAA30
LINC01565
CREB5
LLGL2
TMEM33
LINC00261
CACTIN
MAPK15
ZDHHC2
GMCL1
CDV3
ARHGEF16
LACTB2

CCSER1
MIR30B
TENT5B
CCDC25
PRRT3AS1
NOS3
AN1
CFC1
SOTOS1
ALGS1
CDLS1
PKS
PFIC2
ALPS
ALPS1A
SMA3
DKCA1
EDSPD1
CSB
FOP
CMNS
MYHRS
AEXS
HALP1
FPHH
HH2
KIDAD
PPKP1A
PPKS1
AFD1
MASTC
MASTSYS
MEL
ATXPC
NPS
CMC
PDB3
THIOT
GISTPS
RA
SRS1
XLANP
CGDX

HIGM1
CMTX4
UVSS1
FANCE
FPDMM
NETH
PRIMS
SDS1
PPSH
THC3
TTP
CHNG1
USH3A
WVS
SCIDX1
FANCB
FANCF
PN
MCPH4
HYPT7
ARWH2
HALD2
WAGR
NDNC4
AIMAH1
RDEB
SHEP1
FANCC
HIES2
SCA21
CMM3
FANCJ
NS3
HMPS2
NFTC
CRMCC1
APL
BMIQ14
DBA7
HDLCQ12
ECCL
GLM2
GLM3

FHL5
BRGDA7
ATFB16
MCSZ
D2HGA2
C9D
DKCB2
DKCB3
FANCG
FANCL
SCAR12
JR
THCYT3
CSS2
CMD2B
AOVD2
LPFS2
D2L2AD
VEL
FANCQ
CFC4
BMND17
IMD14
HFE5
FEPS3
BBS16
ANALBA
RJALS
TTD2
TTD3
FANCT
DFNB97
CVID13
PCLD2
NPCA3
MVA3
SPGF28
BMFS5
OCBMD
EV5
NS11
IDDHBA
LPAQTL

NSDVS1
NSDVS2
ARVD14
NS13
OFC1
LMPHM5
HFM
PCOS1
WT4
CNC2
WT3
GLM4
CMM4
LTL
NBLST4
FL1
GLM6
NAFLD1
NBLST7
MIC7
NF4
HPV18I1
LALL
ACO1
ACTG1
APRT
ACTA2
ST3GAL4
PSEN1
ANG
APOE
NR2F2
ARRB1
GRB2
ATP2A2
BCL3
BTG1
HSD17B1
CEACAM1
MS4A1
BMP2
RECE-BMP2
BMP4

CAD
CDH15
CNR1
CBR1
CUX1
CDR2
CGA
CHGA
PSP
CLTC
COL4A3
COL6A1
COL18A1
CSF1
CR2
CRYBB2
ATF2
CARS1
VIPR1
FLI1
EWSR2
EZR
CYP2B6
CYP2E1
CYP4B1
COX6C
DPT
ZIC1
PURA
CSNK1A1
PRDX2
NME3
BPTF
EPHB3
RFXAP
CX3CL1
DFFA
ASCL2
GPI
MTHFD1
SERPINF1
OAS2
TNFRSF6B

MSH5
WISP2
ZNF169
FZD6
LPAR2
SPO11
SPINT1
TLE4
CXCR6
ZNF278
ENC1
TM7SF3
GOPC
TOX
GORASP1
HIPK2
ACADS
BCL2L10
CERS2
GAGE2A
GAGE2B
GAGE12C
GAGE12D
GAGE12E
GAGE12F
GAGE12G
GAGE12H
GAGE12J
GAGE13
GAGE2D
GAGE2E
GAGE10
XAGE3
MAGEE1
MAGEE2
MAGEB10
MAGEB16
MAGEB17
AFF2
FRAXE
DSG2
CYP24A1
ARNT

ERCC1
EEF1G
TYMP
EGF
EPHX1
FPGS
GRP
TACSTD2
GATA2
GFAP
GOT1
ENPEP
GSTA1
GUK1
GNAS
GNAS1
GNB1
GZMA
HBB
HSPG2
HK1
HIST1H1C
H2AFZ
HLA-DRA
HLA-G
HOXC13
HOXD11
MNX1
MIC6
FCGR1A
ADAR
IGHV@
KLK2
LGALS1
AL-A1
LYL1
STMN1
ALOX5
ALOX15
LCP1
YBX1
MPI
CD63

MBD1
MAPT
MUC5AC
MYBL1
MLLT1
MLLT3
MYH11
NDUFV1
HMGB1
NFKBIA
NFKB2
CRK
CTTN
FOS
FOXG1
OSM
SPI1
MECOM
WNT3
REG1A
PPY
ELAVL4
PBX3
PSG1
PRNP
PRDX1
PTGS1
COX3
POMC
PSMA4
PTPN6
PTPN7
PPP2CB
MAPK1
BCHE
SFTPC
RHOG
RAB6A
RALB
RAG1
RAG2
KDM5A
RRM1

RPS6
RPN1
RLF
TSPAN31
SRI
MMP3
SDC1
TLN1
CD247
TRAC
TCL1A
TAL1
TIMP2
GTF2E2
PDGFB
CNTN2
ZFP36
TPM1
TM4SF1
EFNA1
U2AF2
RPS27A
CMPK1
MIR508
MIR509-1
MXRA5
CSAG1
EOLA1
GATA1
IL2RG
SSX1
CD99
TBL1Y
SRY
MTND5
CACNB2
IFI27
CYB561
CDH11
RAD23A
LRP2
TNS1
ADH7

MAP3K10
AP1B1
ASIP
GCH1
ALDH1L1
ID3
HNF4A
AADAC
BTC
RPS8
MEP1B
GSTT1
PRKCQ
RPS3
NTRK2
IK
IL15
REST
ID4
NKX2-5
TGM4
GABPB
GABPB1
NBL1
PPP5C
MPP2
TFAP4
ELOB
LAMA3
DDB2
MRE11
HSPA8
VSNL1
TAGLN
ANP32A
GCKR
PDCD2
GFI1
CDKN2D
GHR
GHBP
GNAQ
PKN1

CTNND1
PDCD6
MFAP5
CDH5
CYP2C9
CAPRIN1
DNAJC3
PTGFRN
PTK2B
MSRA
DDX1
C1QBP
GUCA2B
RHEB
TBXT
KAT5
PLIP
TCEA1
NR2C2
ABCC9
ATOH1
STX1B
BAG1
ZNF146
GRB7
GRB10
CASP6
RND2
ADD3
CCNG1
PTP4A2
PTP4A1
TFAP2B
ABCA3
TBX3
UBE2I
TGFBI
TEAD4
STXBP2
SMARCC1
MGAT5
USP11
ZMYM3

FGF13
USP9X
NONO
MAGEB1
MAGEB2
MAGEB3
MAGEB4
GPC4
OFD1
SSX2
MAGED1
EGFL6
IKBKG
SPANXA1
PPP2R3B
ABCD1
RLIM
CTPS2
SH2D1A
PDHA1
P2RY8
SSX7
SSX8
SSX9
MAGEH1
MCTS1
BRCC3
SPANXN1
SPANXN2
SPANXN3
SPANXN4
SPANXN5
06-Sep
BEX4
BEX5
MIR223
FUBP1
RIPK1
CRADD
RPS19
NFS1
AAMP
LRP6

NMI
MBD3
TRPM1
CDC20
PSIP1
FOXH1
RPS12
PTCH2
RPS23
RPS28
RPL9
04-Sep
RPL6
RPL26
SLIT2
TPD52L2
CCNE2
CDYL
RECQL5
NR1H4
PDZK1
TSSC4
ANGPTL1
TIMELESS
EIF2S2
EIF3H
EIF4G3
FMO5
RASGRP1
AKAP9
CCNA1
NEK2
09-Sep
TTK
ADGRG1
RASAL1
SVIL
ESPL1
RPL38
SELENBP1
SLC22A4
KRIT1
ING2

DBF4
PLXNB2
APPL1
B4GALT7
SSRP1
RAB3D
LMO7
GPNMB
GFI1B
SCGB2A1
KLK7
ACSL6
BAMBI
ITSN2
CBX5
AMACR
LARS2
TONSL
MGAT4B
AIM2
EHMT2
SLC30A9
MGAT3
MGAT4A
VAT1
TLX3
AKAP11
RBM39
ANGPTL3
LILRB2
PUF60
MALT1
MMP24
PCSK7
MTHFD2
CNOT3
SOX5
BRAP
SLC9A3R1
FXYD3
DOK2
ANGPTL2
SENP6

GALNT7
PMS2L1
TERF2IP
WNK1
USH1C
RAD18
NDRG1
CAPN10
PFKFB4
IL22
ALKBH1
MFHAS1
MYCNOS
MINPP1
CXCL16
ABCB6
MMP26
B4GAT1
NRG3
HMG20B
ADAM15
FBXO8
FBXW11
BTRCP2A
TRIM13
BICRA
NOP53
RIPK4
JAM1
DCDC2
DLEU2
TRIM33
FGL1
MAPRE2
PES1
HERC2
B3GNT3
B3GNT4
SEMA6A
PCNT
SART1
PPP2R1A
KIF1B

RNASEH2A
IFT122
DNAJC12
RBM15
SIGLEC12
SESN1
KLK14
RASAL2
CGREF1
CGRRF1
FZD8
FADS1
TNFSF9
SLC45A2
SELENON
MSRB1
NUDT6
NCAPG
RAB38
DDX43
WNT3A
WNT8A
WNT5B
ST6GALNAC4
ATF5
ELMO2
ZNF320
HTRA2
SS18L2
MARK4
UGT2B28
HUNK
CLIC4
SLC36A1
RAI14
BAALC
FBXO32
PYY2
HAVCR2
TIMM8B
NCKIPSD
NSD1
NAT8

OSBP2
OSBPL5
OSBPL10
IL17RD
CITED4
NAGK
FXN
KMT2B
CST6
TRAF2
TRAF3
ZNF148
SUMO1
ATP2A3
WNT7B
WNT2B
CCDC6
LIMK2
NOVA2
GPR18
GPR31
RAC1
RAC2
NINJ1
NRP2
YEATS4
MAPKAPK3
MIA2
PSMB4
ELF3
RREB1
ZMYM2
EIF4EBP1
EIF4EBP2
SOX10
TNFRSF9
MFGE8
OVOL1
SEL1L
ABLIM1
EMP1
TRAF6
UPK1B

HAND2
BRPF1
FADD
TRDMT1
CYTH2
TFG
LGALS4
RNF217AS1
DJ1
CELF2
KIF2A
CXADR
FHL2
MCM4
TUBB3
MARK3
NCOA1
PRKAA1
HIST1H4I
CHN2
WNT9A
IGFBP7
GPR19
MARCKSL1
TADA3
PRMT1
CDH6
CDH8
CDH19
CDH17
MNT
CHEK1
USP12
TCF20
UROS
ELP4
SIMO
ACADM
SMG1
PI15
NSD3
APOBEC3G
RPTOR

VENTX
DYNLRB1
DYNLRB2
CRELD1
PDZK1IP1
ST3GAL1
PTF1A
PUM1
CARD10
CARD14
CARD9
IRF5
APOL3
MORF4L1
LOXL4
SFMBT1
ALPK1
TLN2
AIRE
GLYAT
ENOSF1
EPSTI1
SBDS
PPP1R13L
PARD6A
MAML2
ARHGEF2
SMPD1
ANGPTL5
SAR1B
SAR1A
LCOR
C10ORF12
TJP2
SARM1
USP32
APOBEC3C
CTNNBIP1
SESN2
PLEKHA4
PLEKHA5
PCSK9
TET1

PHF11
OTOP2
NKD1
SETDB2
SPRYD7
RCBTB1
CMTM6
TRIB3
NTM
CEP57
RANGRF
DHX32
SEMA7A
SPRY4
SPOCK3
ASH1L
HIPK1
GALNT10
CYP2A13
ERRFI1
ELAC1
MRO
NUAK1
NUAK2
CENPV
SPDEF
PTGES2
PRSS21
SEMA3E
DDX41
EXT2
IRGM
ADSL
GALNT14
CCNB1IP1
PAXIP1
RRAGC
SLC28A3
CHST15
IGF2BP2
STOML2
ARG1
PRPF19

DCXR
GSDMC
ETHE1
CDKL3
ANAPC1
MMP25
PRICKLE2
CYBA
LAMTOR5
FBXO38
PXRICS
HMCN1
CTDSPL
RAB11FIP2
NBPF12
C1ORF152
JDP2
ANO6
CREB3L2
FGFR1OP2
POTEB
POTEH
POTEE
POTEA
POTEG
ARL5A
VASH1
RND1
POU6F2
AJUBA
KDM2B
APITD1
CLDN7
EEF1E1
WIPI1
NUDT5
SPRED1
TRIM36
OIT3
ANGPTL6
NLRP2
CDCA5
PHLPP1

SYNJ2
MIR19A
MIR19B1
MIR20A
MIR92A1
MZB1
MRTFB
CYP27B1
RASGRP3
ATAD5
ARHGAP20
MIR122A
GOLGA8B
PDGFD
OS9
MAL2
CDCA7L
PSMG2
MIR24-1
miRNA189
LPL
YPEL3
CCDC88A
CADM4
ACOX1
NCOA7
NKAIN2
AMICA1
UBN1
API5
UBAP1
NEK8
DCP2
ETNK1
ARHGAP21
CRYL1
KAT7
UBR4
SPIN1
CDCA7
HES2
ARSJ
VPS45

SARNP
CHMP3
H2AFY
MYO18A
RINT1
ANO1
TSPAN33
CHST11
ST8SIA6
IGF2AS
CENPM
INO80
CEP162
AKT1S1
RPS19BP1
DERL2
RNASEH2B
RUFY1
RUFY2
RNASEH2C
PHF20
P3H1
EPB41L4B
LINS1
SLC2A12
TMPRSS11E
NTN4
RMI1
SPTSSB
SAMD9
LOH3CR2A
LRRC4
DCAF6
BRE
PAF1
FAM126A
RASSF4
PRSS53
ZC3H12A
ZNF687
YTHDF2
SMYD2
PHB2

USP28
UST
BZRAP1
CHD5
DRAM1
SLC25A33
MEX3A
MEX3B
TMEM259
RGSL1
RGSL2
TMEM30B
LUZP6
PPFIA1
SETBP1
NELFB
ANKRD11
UNC45A
GSDMB
CLDN8
CHCHD7
KIF27
NOXO1
KLHDC2
KCTD5
ABLIM3
SCARA5
C9ORF64
C9ORF103
RASEF
GKAP1
CNTROB
MTHFD1L
TTC21A
WDR46
GLB1
EBP
PLEKHM1
GATA5
KDM3A
POLDIP2
12-Sep
13-Sep

BANP
CKAP2
MIR206
PLA2G12B
ADAMTS20
HIPK4
TCTEX1D4
SCUBE2
VMP1
STARD3NL
MIR128-2
MIR128-1
MEMO1
CMPK2
LCORL
PALI2
ARMC10
OTUD6B
GRASP
APC2
RASL10B
IDO2
ECHDC1
MIRLET7A2
MIRLET7C
MIR26A1
MIR26B
CAB39
MYSM1
RGL4
ATAD3A
CCDC34
MIRN610
LIN7C
GGT7
RASL11A
FAT4
RAB24
ARFGAP3
GEN1
HBS1L
ARID3B
METTL3

TIPARP
RNF31
ABCC10
MIRN101-2
MIR370
RAB1B
RNMTL1
WRAP53
RERG
RAB10
VTRNA1-1
VTRNA1-2
VTRNA1-3
CCNY
UTP20
TAF1D
DHRS3
QSOX2
LANCL2
RAB4B
RAB22A
DEPDC6
SDHAF2
CTC1
MIR184
MIR184*
POLN
FAM102A
ENOX1
RBCK1
TWF1
CRNKL1
SMG6
TRAPPC1
ETAA1
TAOK2
TMEM18
ZNF296
C18ORF54
SLX4
SPATA13
KIAA1549
ARHGAP18

DRAM2
DDX42
MIR138-1
ADI1
MIA3
HPSE2
SPEN
ATG14
RUBCN
NTMT1
ALDH1L2
BTN3A1
ATF7IP
ACKR1
MIR130B
EIF2D
CCDC115
ZNF260
SCAMP5
RNF213
TMEM205
PLAAT3
BAHD1
TOE1
TMEM25
CDC42BPG
NBPF9
KPNA7
TENT2
SPECC1L
DIS3L2
CHTOP
SLC16A9
ZNF638
TDRD3
TFAP2E
C9ORF116
KLHDC8A
BRAT1
HIF1AAS1
RASGEF1A
ZDHHC7
ZDHHC21

LINC00538
FAM123A
RPRD1B
RASSF10
CEP63
MMS19
TMEM207
MIR199B
TMUB1
SH2D4A
GATAD2A
GATAD2B
MIR410
MIR487B
GOLT1B
ASUN
TUBB2A
PRSS55
PGAP2
DHRS4L1
DHRS4L2
MIR1909
MIR1915
IFNE
RC3H2
EXOC8
CCDC28A
GNG12AS1
CDK12
MIR297
TIMMDC1
ARMC5
SPAG5
CHCHD6
SH3BGRL3
ZNF451
BUD23
DELE1
CEACAM8
TNFAIP8L1
RSL1D1
PPP2R3C
MIR520C

BTBD10
MAGI3
MIR99AHG
MIR100HG
MYCNUT
MIR339
TSTD1
SPRTN
UQCC3
IFIT5
RNF220
DCUN1D3
DDRGK1
CMTR2
ZBTB49
CLEC4G
MIR520G
FRMD4A
LINC01018
TAX1BP3
EWSAT1
SFR1
DDX31
DAPK2
FAM118B
FAM30A
SNORD13
SKA2
ZNF589
TAOK3
NIT2
GCNT4
IGDCC4
NCOA5
WDR83
TMEM14A
MRFAP1
CCDC68
DHRS4AS1
CHD9
PDIA5
GIMAP6
ABHD6

MIR490
NORAD
DNAJC21
TSR3
LINC00673
RNASEK
TFAP2AAS2
HAND2AS1
RUNDC3B
FAM26F
SHF
PRAG1
AGBL2
AGBL3
SHC4
PRRC2C
INCA1
KLHDC9
CNKSR3
IPCEF1
COLGALT1
COLGALT2
LINC00672
TP53INP2
SRXN1
SPRY4IT1
EQTN
TXNL4B
KBTBD7
MFSD12
LIMCH1
BAGE2
BAGE3
THEMIS2
PSMD6
TP53I11
PYROXD2
HILPDA
AEBP2
LDLRAD3
KLHL22
ZNF689
SPZ1

CNOT6L
WDFY1
MPV17L
A1CF
ZNF341
ZNF197
VHLAK
DUSP26
CAP2
BRINP3
PLEKHB2
BRD7
BICRAL
WAKMAR2
PEAK3
ZBTB10
CDH24
HIGD1A
SH3RF1
ZDHHC19
PSORS1C3
ZNF281
EPGN
NEK10
CACUL1
LAYN
ROMO1
ZNFX1
ANGEL2
BIVM
MIR30D
M1AP
