# Supplementary material for: Network Pharmacology-Based Study to Uncover Potential Pharmacological Mechanisms of Korean Thistle (Cirsium japonicum var. maackii (Maxim.) Matsum.) Flower against Cancer
Source: Molecules. 2021 Sep 29;26(19):5904. doi: 10.3390/molecules26195904 (PMC8513069; doi:10.3390/molecules26195904)
Supplement: Supplementary file 1 [file molecules-26-05904-s001.zip › Supplementary Table S3.pdf]

The number of final 51 targets against cancer

AKR1B1

ADORA3

ADORA2A

ADORA2B

IMPDH2

PAM

EPHX1

FABP3

PRKCA

S1PR3

PPARA

HMGCR

CDC25B

PTGER2

CDC25A

PPARG

FOLH1

PTGER4

PTGFR

PPARD

S1PR1

TRPV1

CNR1

KDM5C

PRKCE

VEGFA

TOP2A

CYP17A1

SHBG

NR1H3

ESR2

AR

MGLL

FNTB

PHLPP1

BCHE

AKT1

SRD5A2

ESR1

SHH

CYP19A1

NR1H4

VDR
CYP24A1
PTPN1
PTPN6
IL1B
PTPRF
ABCB1
CA2
CA1
